# Supplementary material for: Activity of Colocasia esculenta (Taro) Corms against Gastric Adenocarcinoma Cells: Chemical Study and Molecular Characterization
Source: Int J Mol Sci. 2023 Dec 23;25(1):252. doi: 10.3390/ijms25010252 (PMC10778756; doi:10.3390/ijms25010252)

S1. ^{1}H NMR (600 MHz, CD_3OD) of *lariciresinol-4-O-β-D-glucopyranoside* (**1**)
S2. HSQC (600 MHz, CD_3OD) of *lariciresinol-4-O-β-D-glucopyranoside* (**1**)
S3. ^{1}H NMR (600 MHz, CD_3OD) of *(+)-lariciresinol-9-O-β-D-glucopyranoside* (**2**)
S4. HSQC (600 MHz, CD_3OD) of *(+)-lariciresinol-9-O-β-D-glucopyranoside* (**2**)
S5. ^{1}H NMR (600 MHz, CD_3OD) of *byzantionoside B 9-O-α-L-rhamnopyranosyl-(1''→2')-β-D-glucopyranoside* (**3**)
S6. HSQC (600 MHz, CD_3OD) of *byzantionoside B 9-O-α-L-rhamnopyranosyl-(1''→2')-β-D-glucopyranoside* (**3**)
S7. HMBC (600 MHz, CD_3OD) of *byzantionoside B 9-O-α-L-rhamnopyranosyl-(1''→2')-β-D-glucopyranoside* (**3**).
S8. ^{1}H NMR (600 MHz, CD_3OD) of *byzantionoside B **(4)***
S9. HSQC (600 MHz, CD_3OD) of *byzantionoside B **(4)***
S10. HMBC (600 MHz, CD_3OD) of *byzantionoside B **(4)***
S11. ^{1}H NMR (600 MHz, CD_3OD) of d*ehydrodiconiferyl alcohol-4-O-β-D-glucopyranoside* (**5**)
S12. HSQC (600 MHz, CD_3OD) of d*ehydrodiconiferyl alcohol-4-O-β-D-glucopyranoside* (**5**)
S13. HMBC (600 MHz, CD_3OD) of d*ehydrodiconiferyl alcohol-4-O-β-D-glucopyranoside* (**5**)
S14. ^{1}H NMR (600 MHz, CD_3OD) of *(+)-isolariciresinol* (**6**)
S15. HSQC (600 MHz, CD_3OD) of *(+)-isolariciresinol* (**6**)
S16. HMBC (600 MHz, CD_3OD) of *(+)-isolariciresinol* (**6**)
S17. ^{1}H-^{1}H-COSY (600 MHz, CD_3OD) of *(+)-isolariciresinol* (**6**)
S18. ^{1}H NMR (600 MHz, CD_3OD) of *dehydrodiconiferyl alcohol-9-O-β-D-glucopyranoside* (**7**)
S19. HSQC (600 MHz, CD_3OD) of *dehydrodiconiferyl alcohol-9-O-β-D-glucopyranoside* (**7**)
S20. HMBC (600 MHz, CD_3OD) of *dehydrodiconiferyl alcohol-9-O-β-D-glucopyranoside* (**7**)
S21. ^{1}H-^{1}H-COSY (600 MHz, CD_3OD) of *dehydrodiconiferyl alcohol-9-O-β-D-glucopyranoside* (**7**)
S22. ^{1}H NMR (600 MHz, CD_3OD) of *3,4,5-tri-O-methyl gallic acid* (**8**)
S23. HSQC (600 MHz, CD_3OD) of *3,4,5-tri-O-methyl gallic acid* (**8**)
S24. ^{1}H NMR (600 MHz, CD_3OD) of (+)-l*ariciresinol* (**9**)
S25. HSQC (600 MHz, CD_3OD) of (+)-l*ariciresinol* (**9**)
S26. ^{1}H NMR (600 MHz, CD_3OD) of *americanol A* (**10**)
S27. HSQC (600 MHz, CD_3OD) of *americanol A* (**10**)
S28. HMBC (600 MHz, CD_3OD) of *americanol A* (**10**)

S1. ^{1}H NMR (600 MHz, CD_3OD) of *lariciresinol-4-O-β-D-glucopyranoside* (**1**)

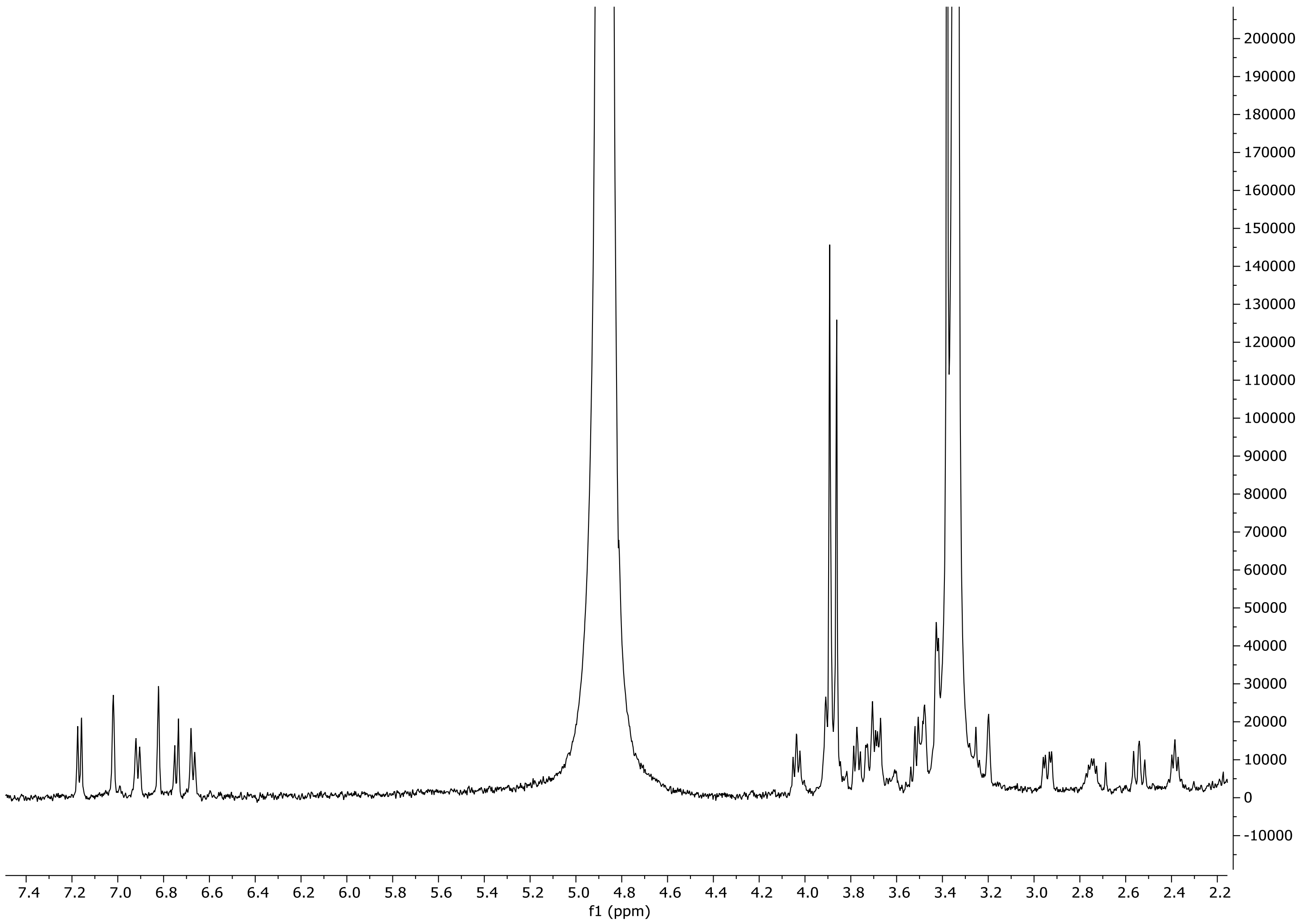

S2. HSQC (600 MHz, CD_3OD) of *lariciresinol-4-O-β-D-glucopyranoside* (**1**)

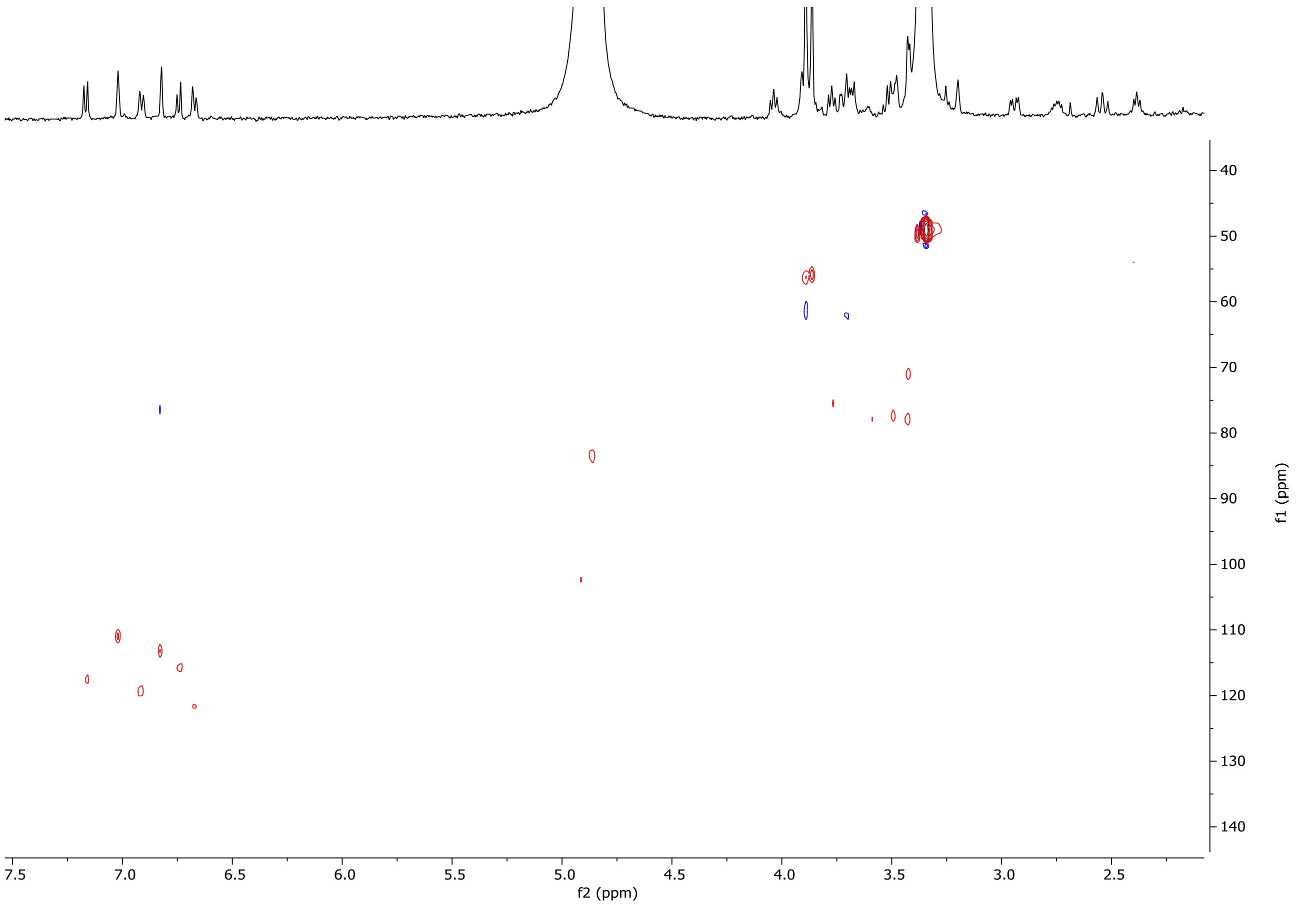

S3. ^{1}H NMR (600 MHz, CD_3OD) of *(+)-lariciresinol-9-O-β-D-glucopyranoside* (**2**).

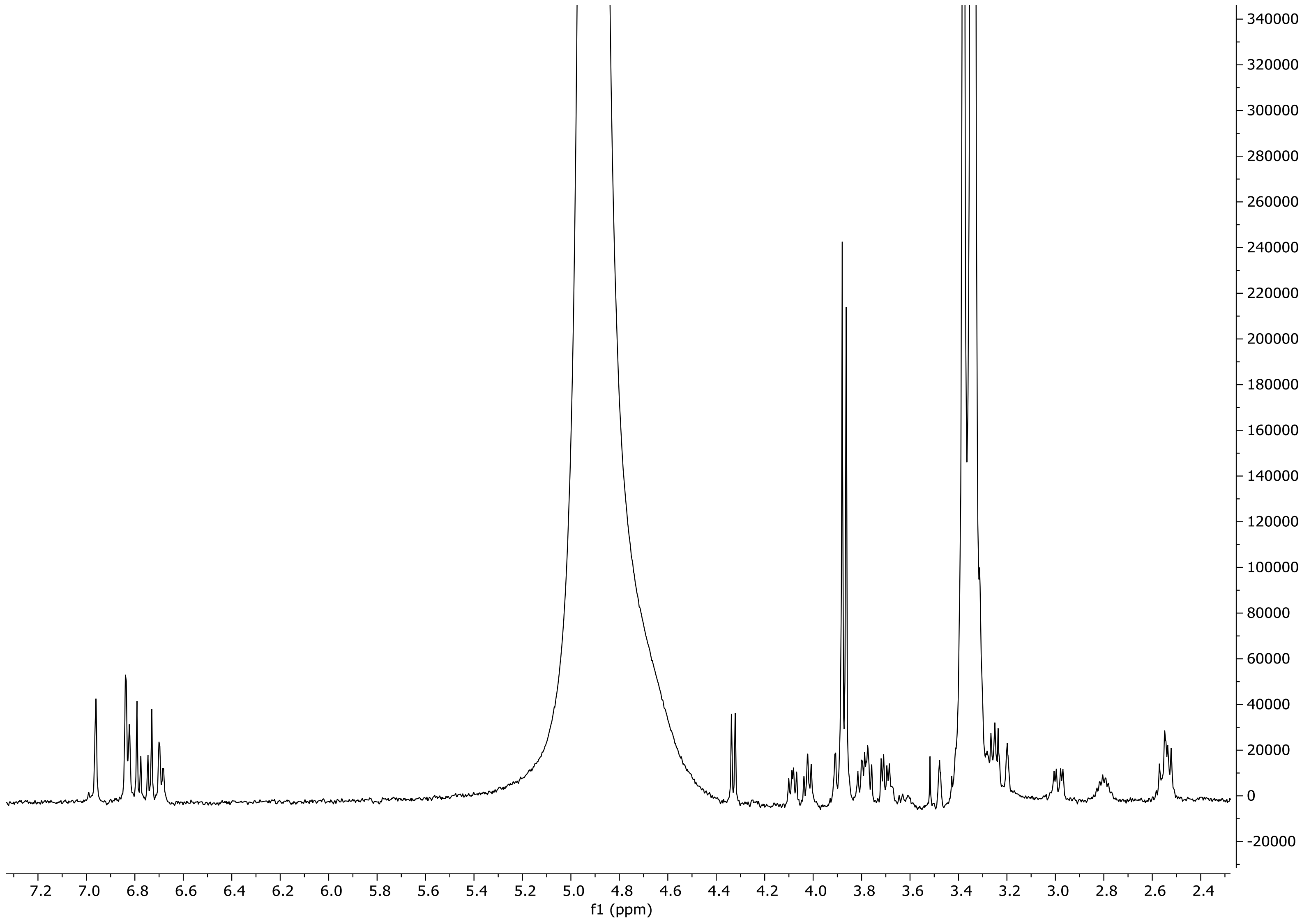

S4. HSQC (600 MHz, CD_3OD) of *(+)-lariciresinol-9-O-β-D-glucopyranoside* (**2**).

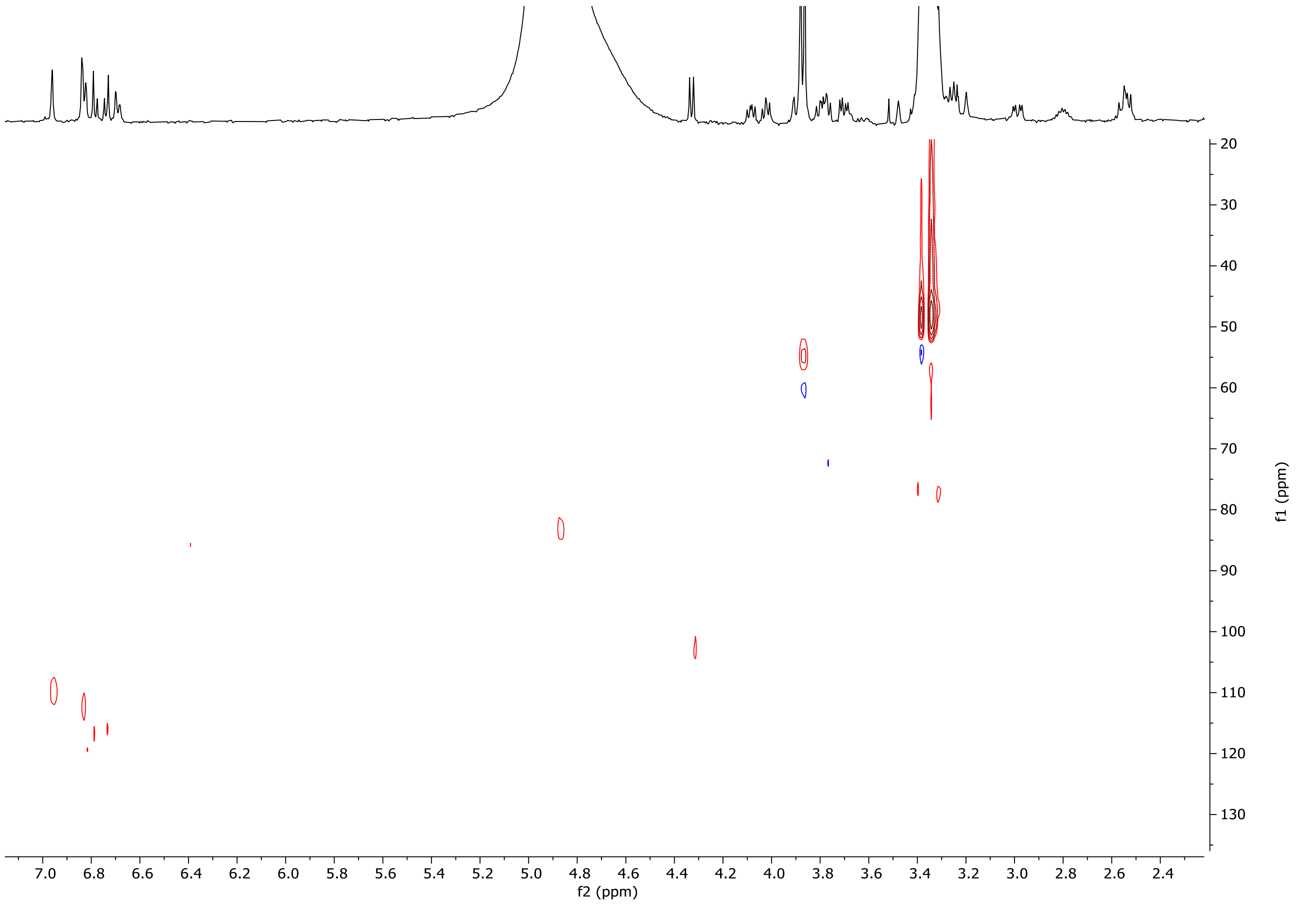

S5. ^{1}H NMR (600 MHz, CD_3OD) of *byzantionoside B 9-O-α-L-rhamnopyranosyl-(1''→2')-β-D-glucopyranoside* (**3**).

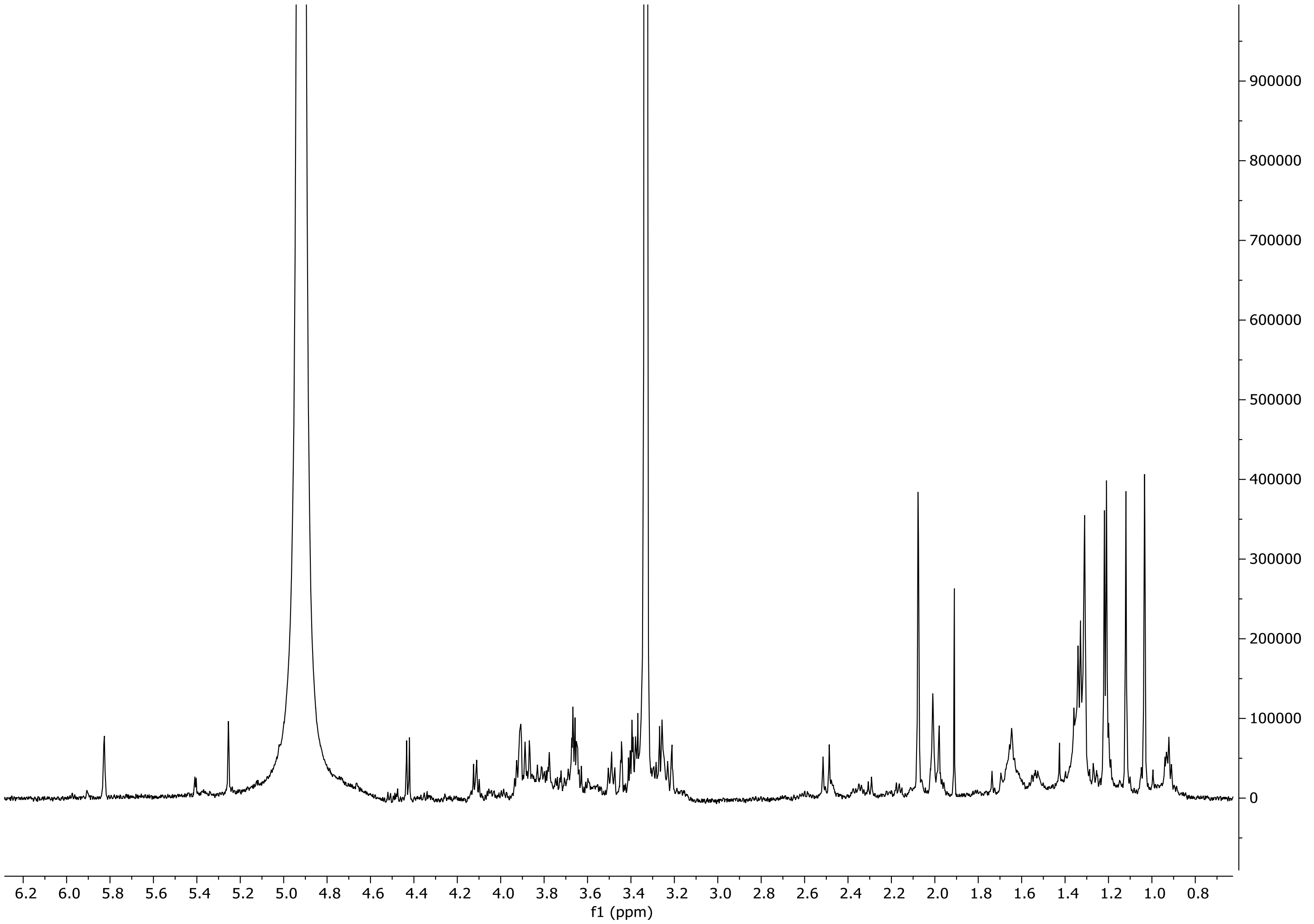

S6. HSQC (600 MHz, CD_3OD) of *byzantionoside B 9-O-α-L-rhamnopyranosyl-(1''→2')-β-D-glucopyranoside* (**3**).

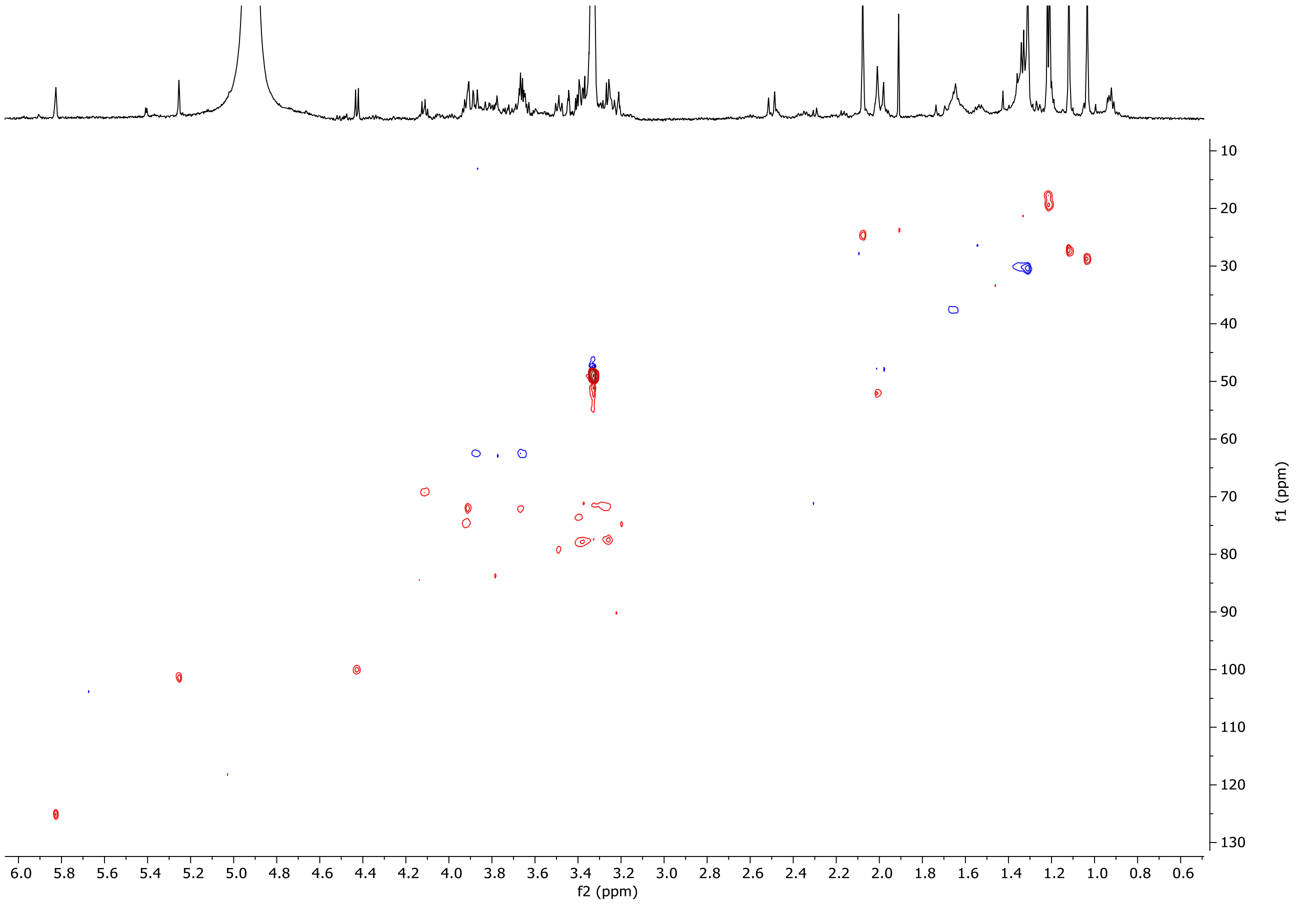

S7. HMBC (600 MHz, CD_3OD) of *byzantionoside B 9-O-α-L-rhamnopyranosyl-(1″→2′)-β-D-glucopyranoside* (**3**)

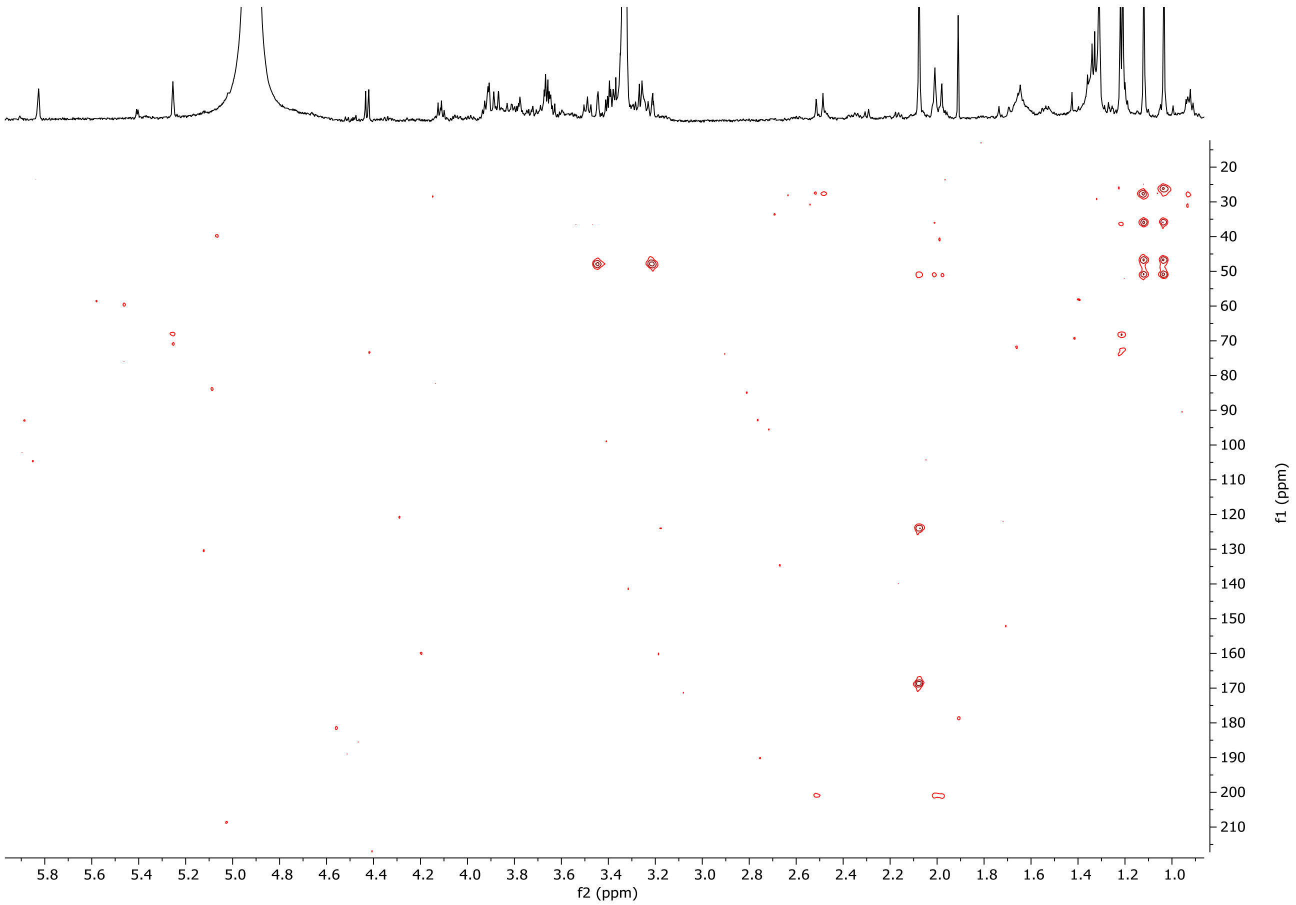

S8. ^{1}H NMR (600 MHz, CD_3OD) of *byzantionoside B (**4**)*.

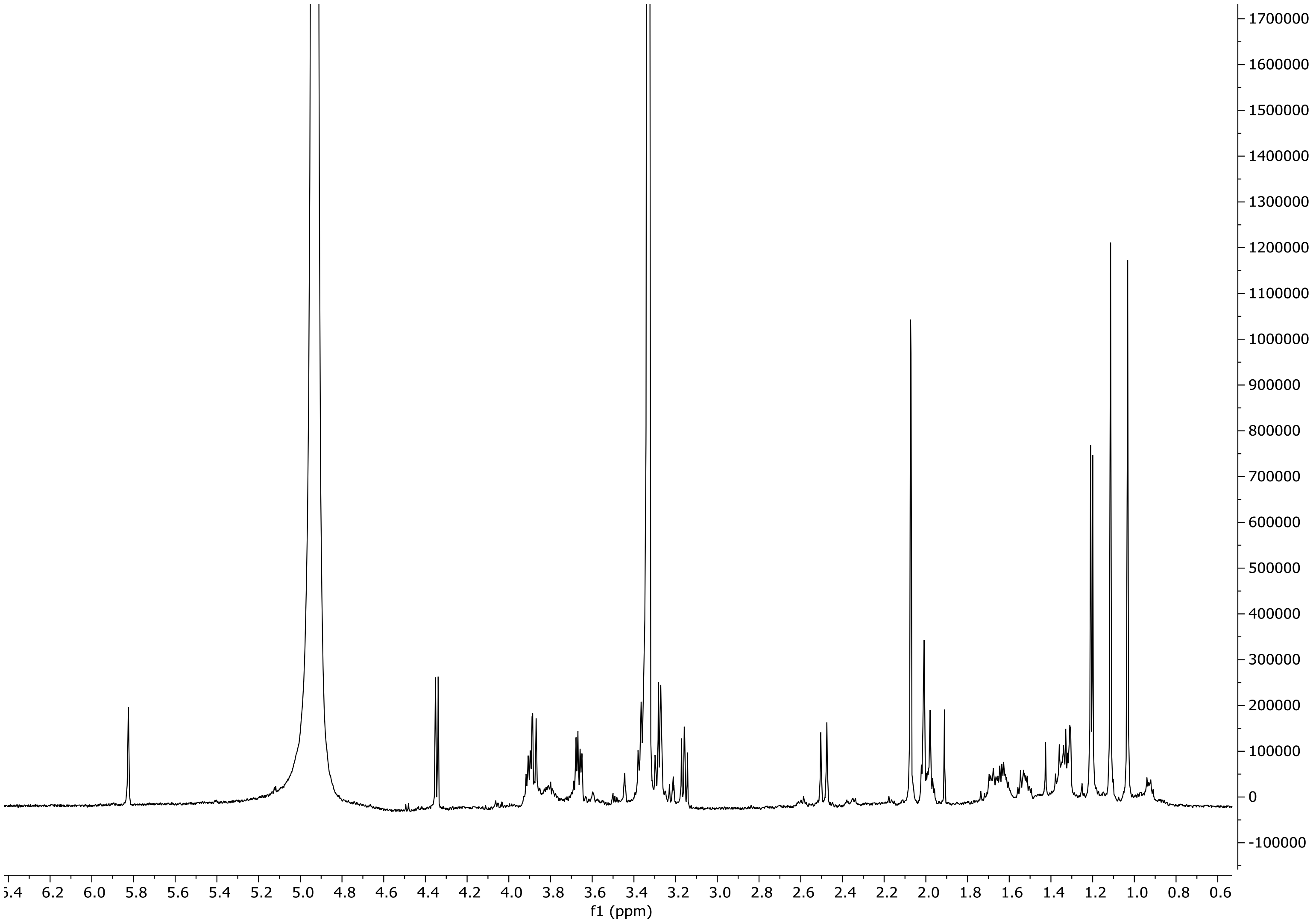

S9. HSQC (600 MHz, CD_3OD) of *byzantionoside B* (***4***).

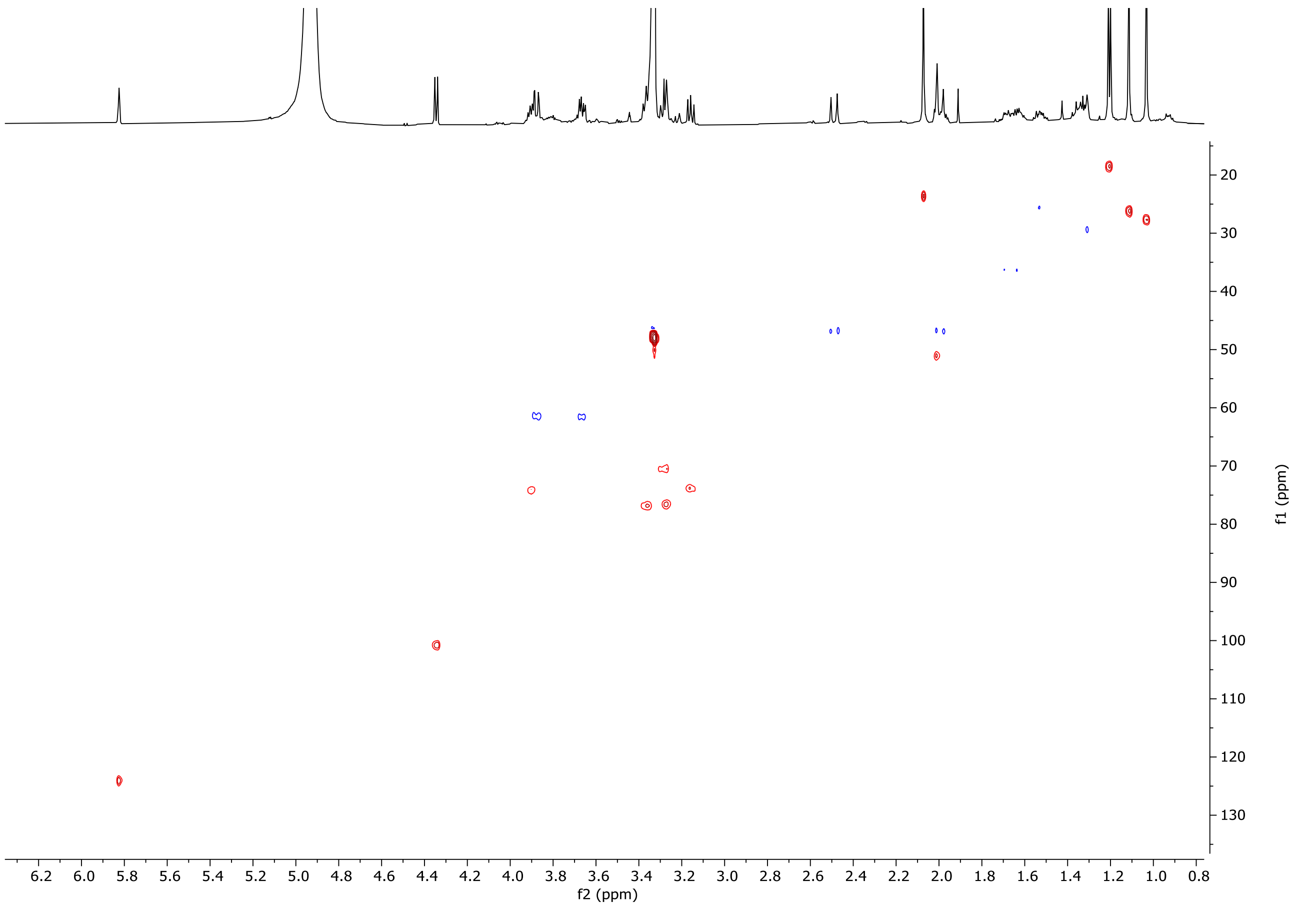

S10. HMBC (600 MHz, CD_3OD) of *byzantionoside B* (***4***).

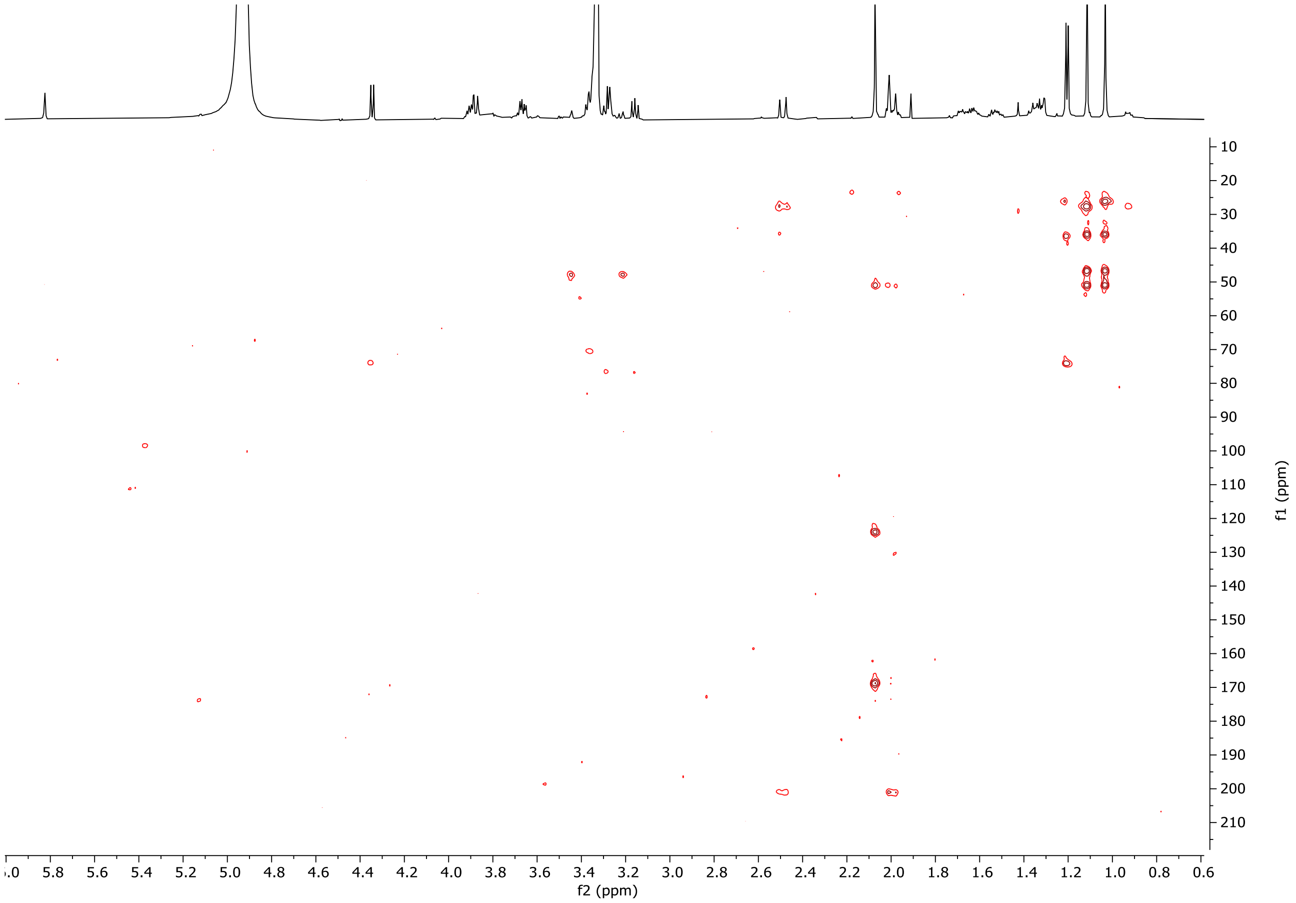

S11. ^{1}H NMR (600 MHz, CD_3OD) of d*ehydrodiconiferyl alcohol-4-O-β-D-glucopyranoside* (**5**).

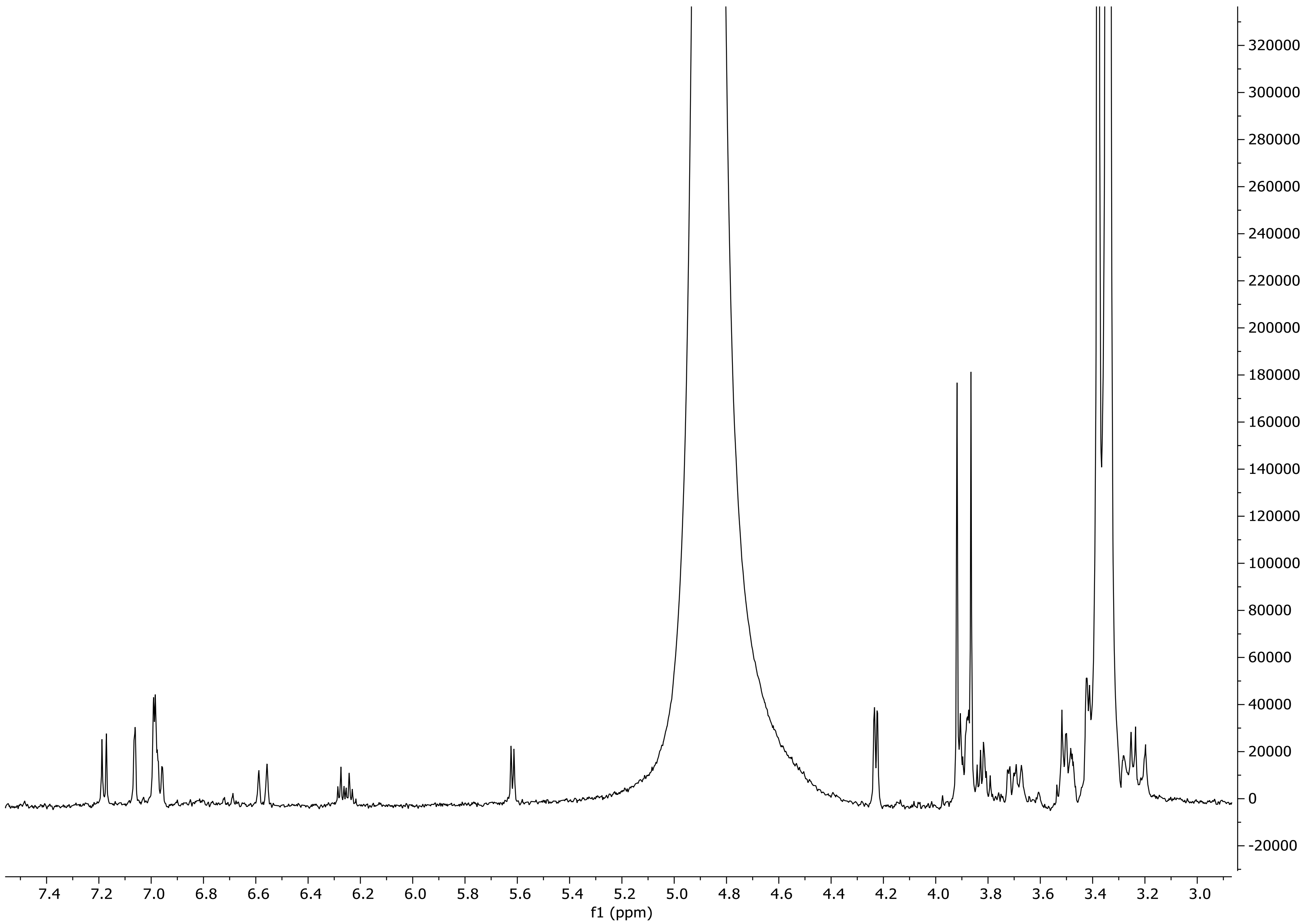

S12. HSQC (600 MHz, CD_3OD) of d*ehydrodiconiferyl alcohol-4-O-β-D-glucopyranoside* (**5**).

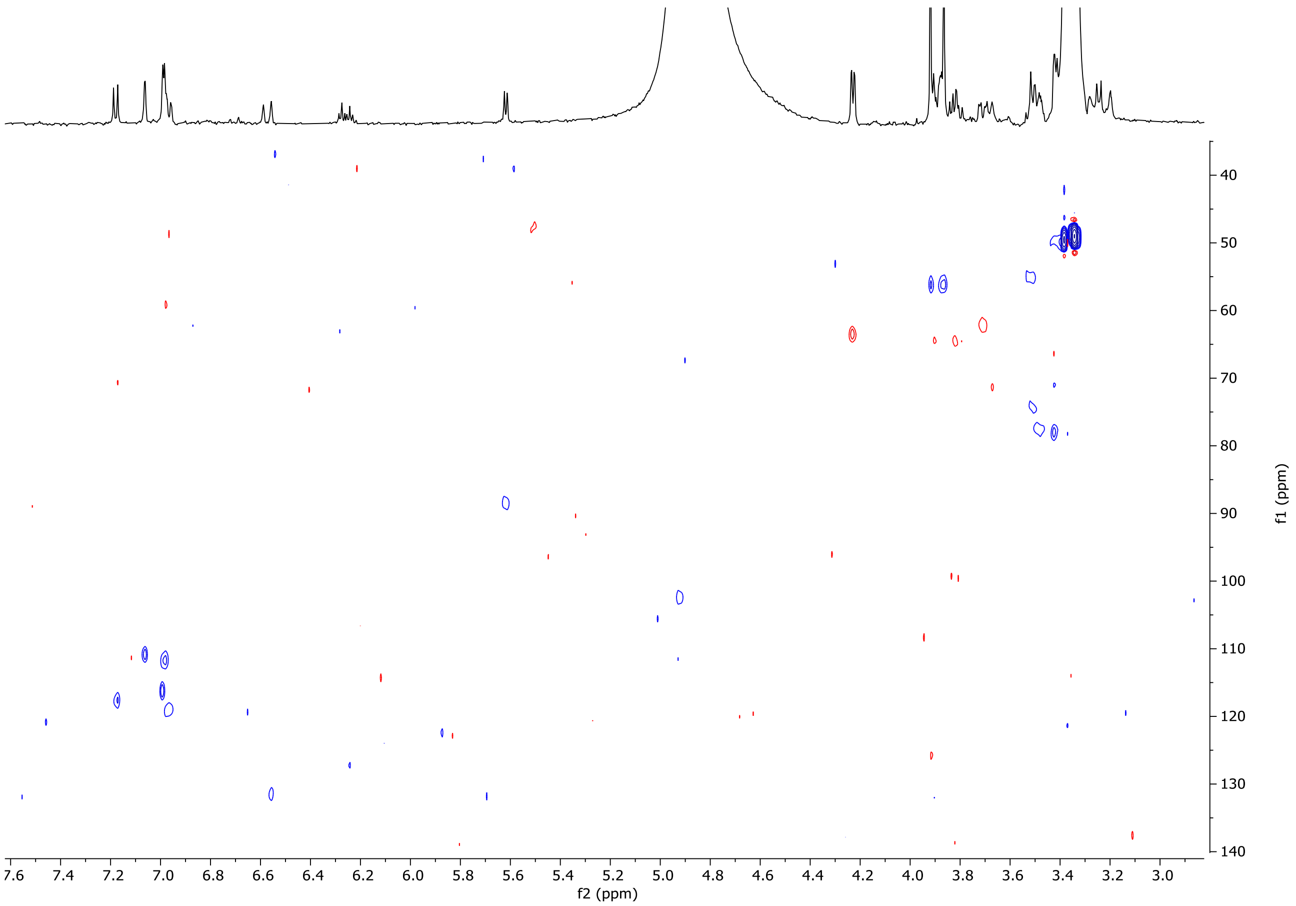

S13. HMBC (600 MHz, CD_3OD) of d*ehydrodiconiferyl alcohol-4-O-β-D-glucopyranoside* (**5**).

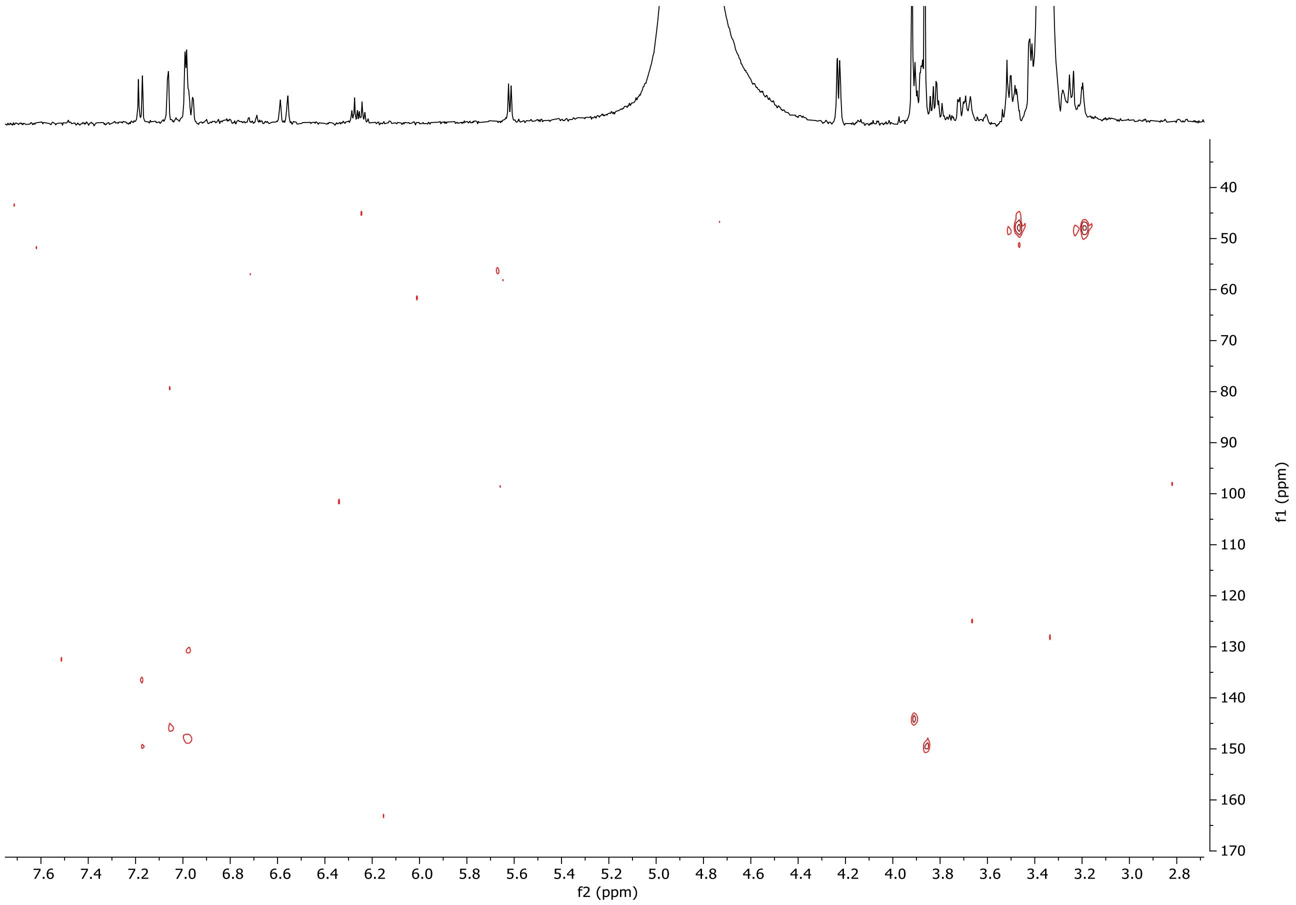

S14. ^{1}H NMR (600 MHz, CD_3OD) of *(+)-isolariciresinol* (**6**).

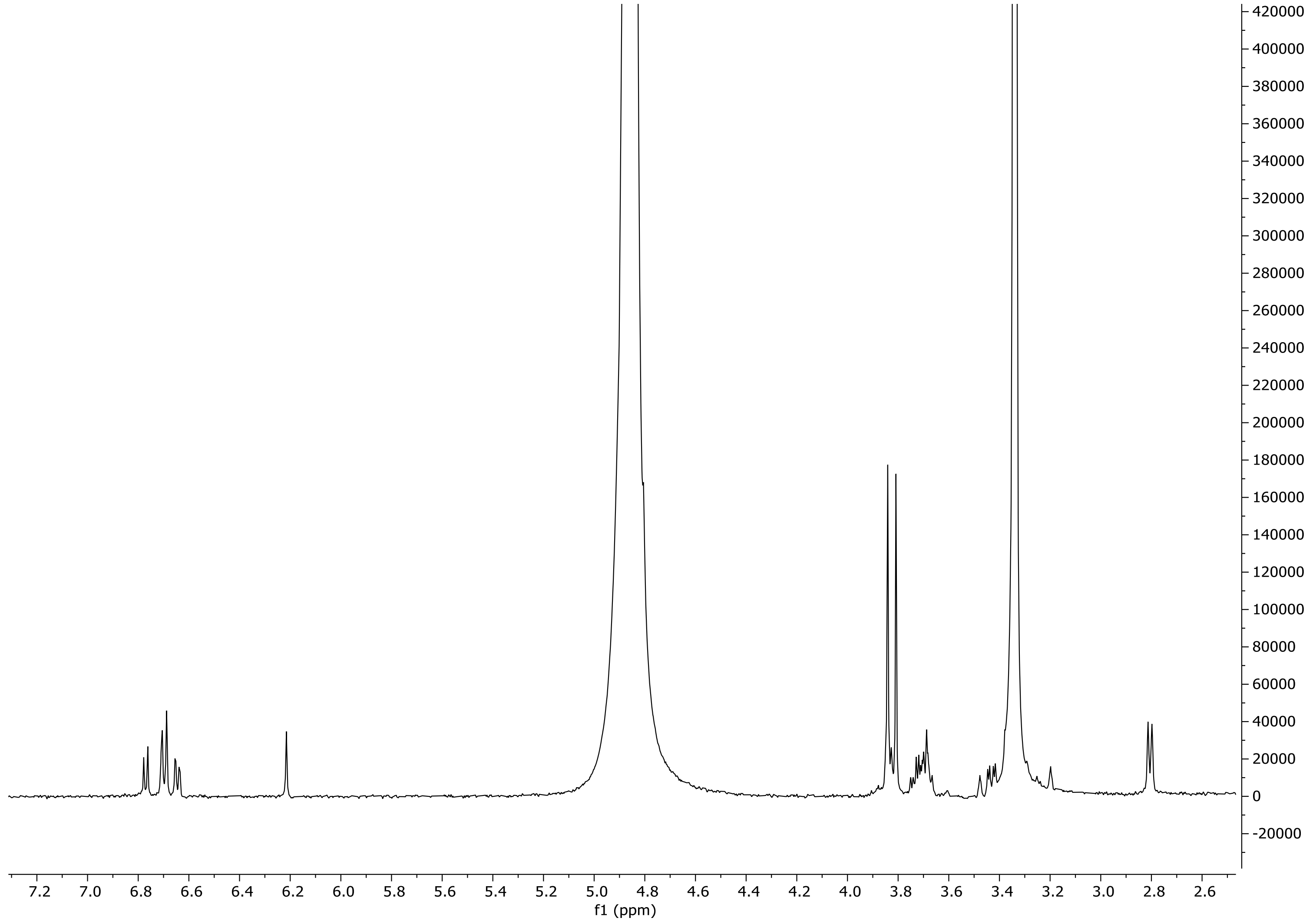

S15. HSQC (600 MHz, CD_3OD) of *(+)-isolariciresinol* (**6**).

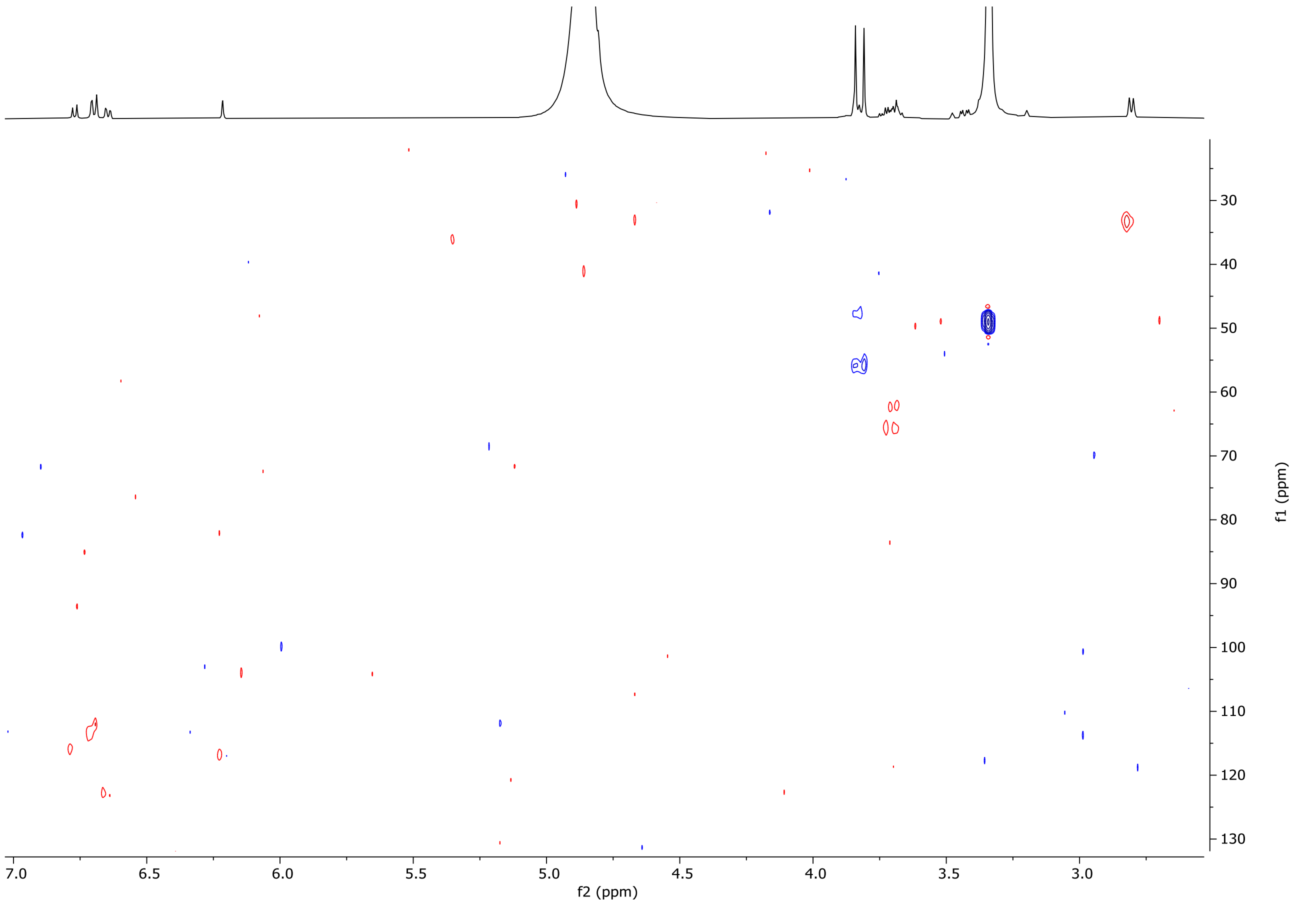

S16. HMBC (600 MHz, CD_3OD) of *(+)-isolariciresinol* (**6**).

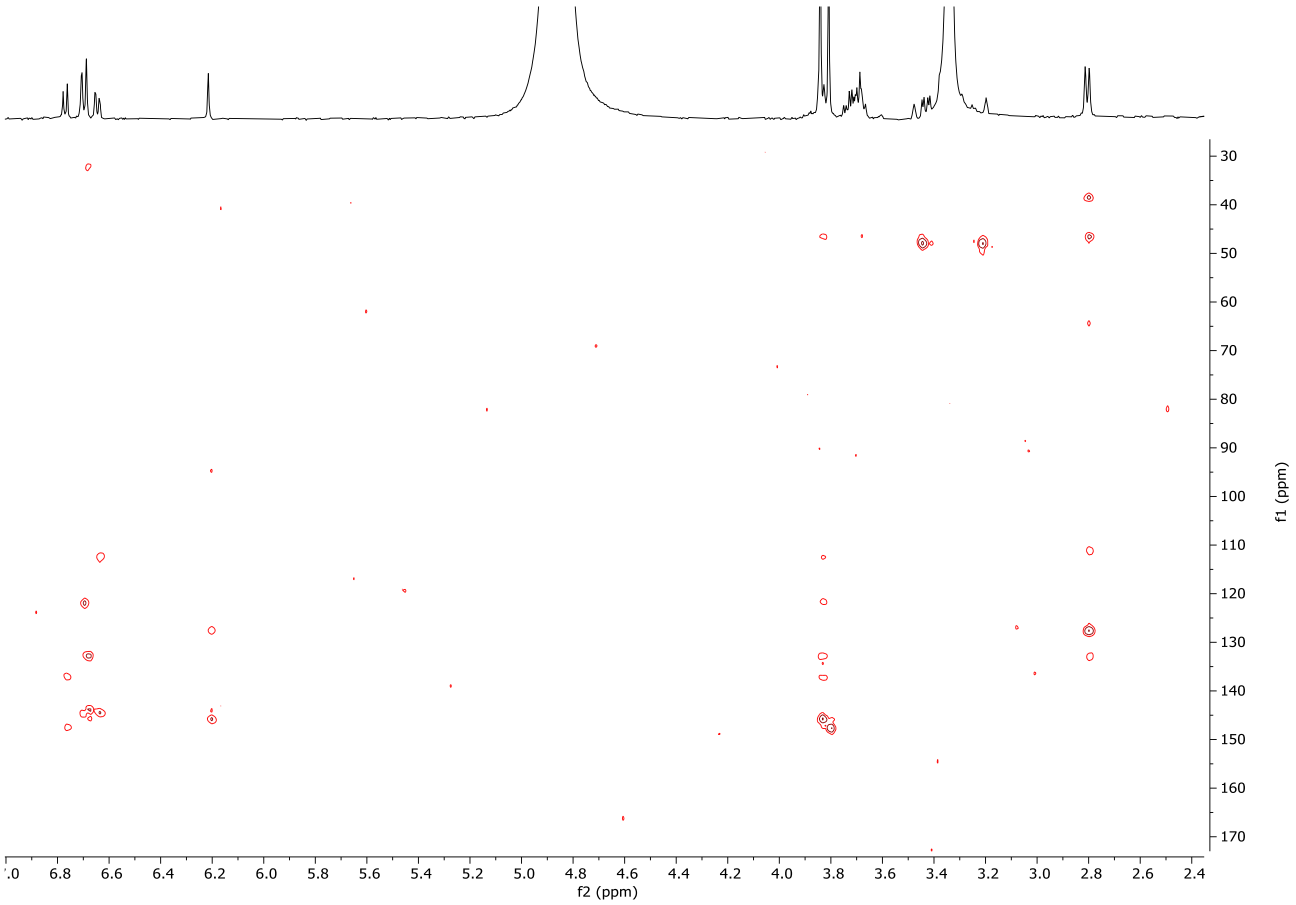

S17. ^{1}H-^{1}H COSY (600 MHz, CD_3OD) of *(+)-isolariciresinol* (**6**).

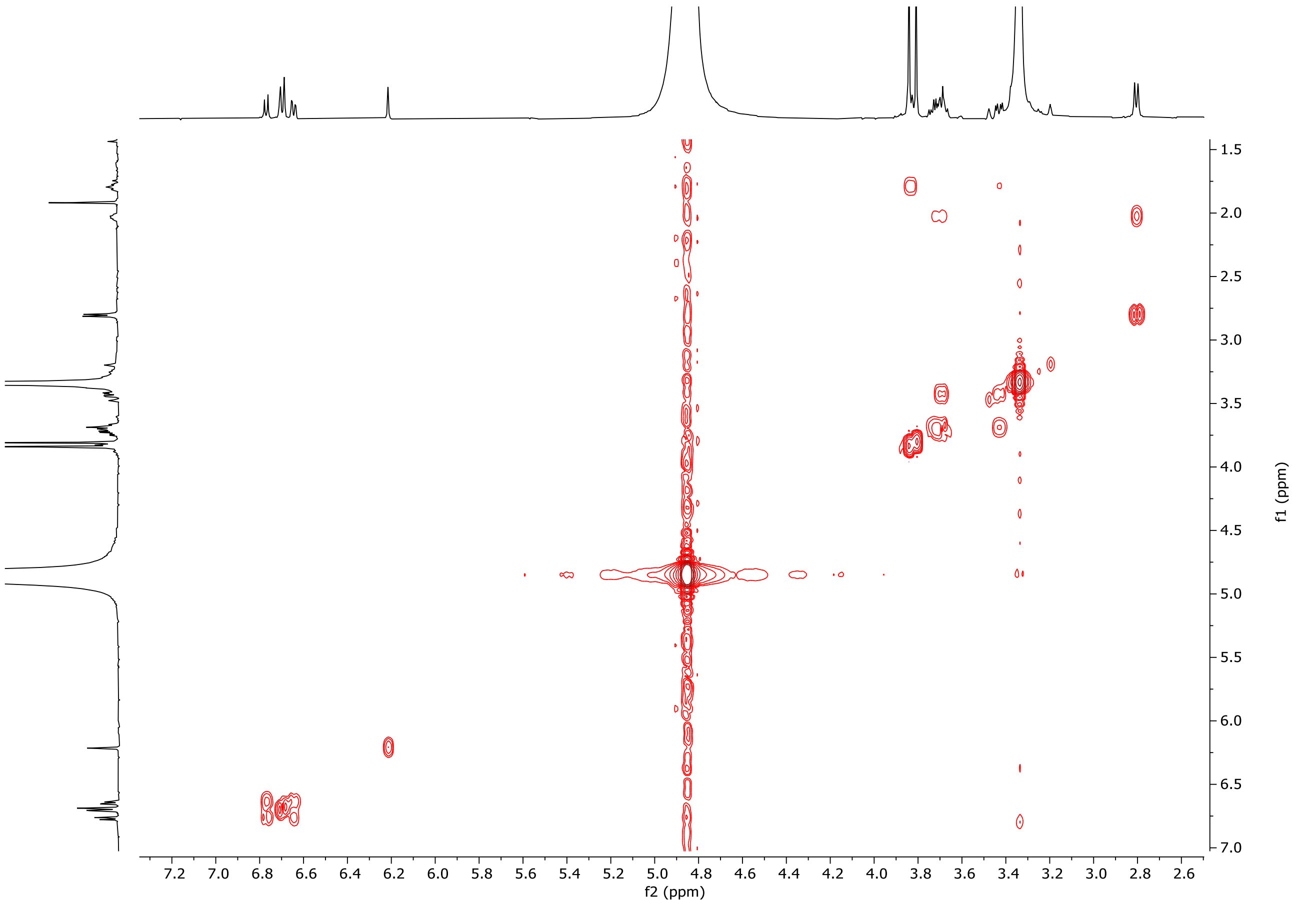

S18. ^{1}H NMR (600 MHz, CD_3OD) of *dehydrodiconiferyl alcohol-9-O-β-D-glucopyranoside* (**7**).

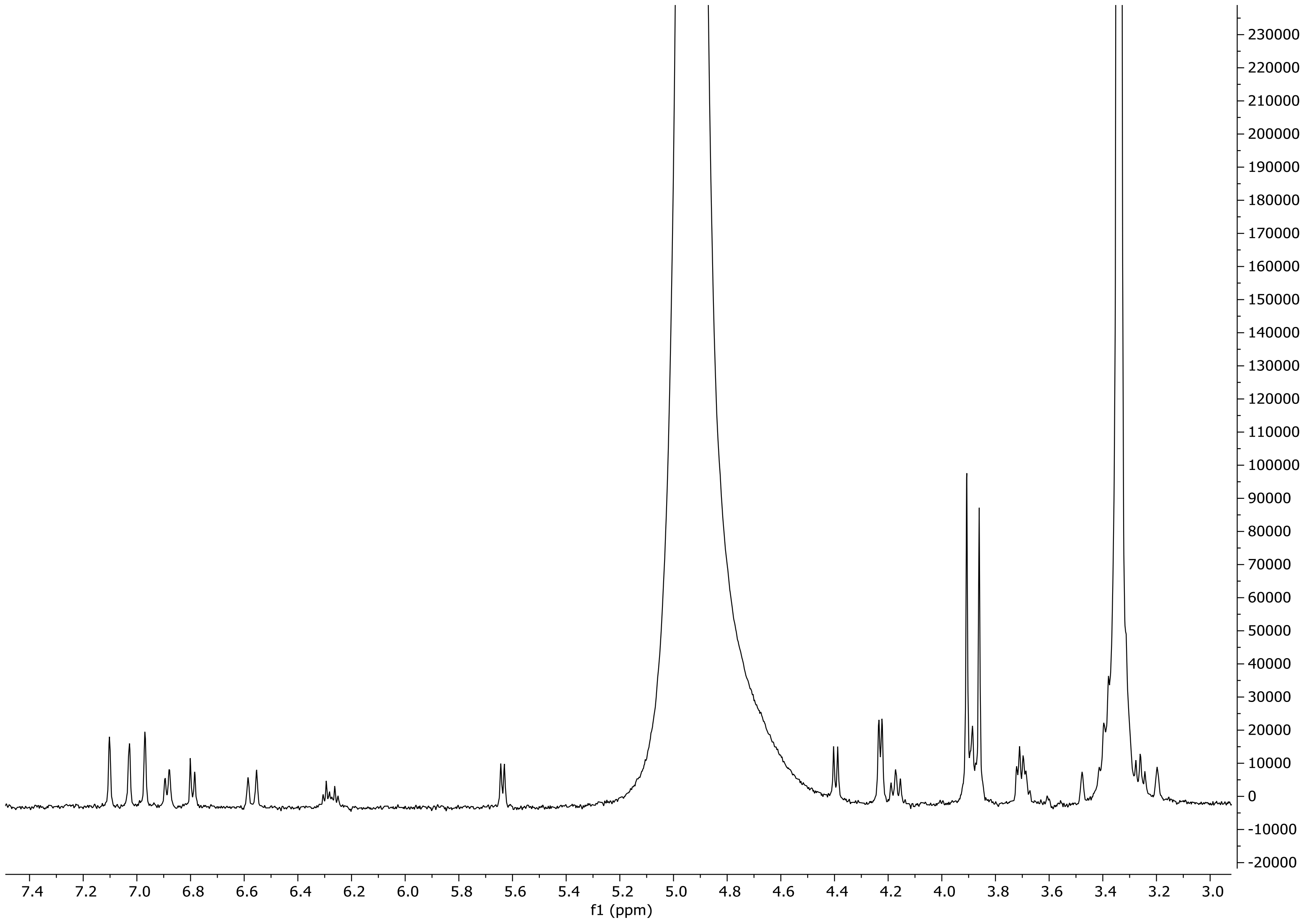

S19. HSQC (600 MHz, CD_3OD) of *dehydrodiconiferyl alcohol-9-O-β-D-glucopyranoside* (**7**).

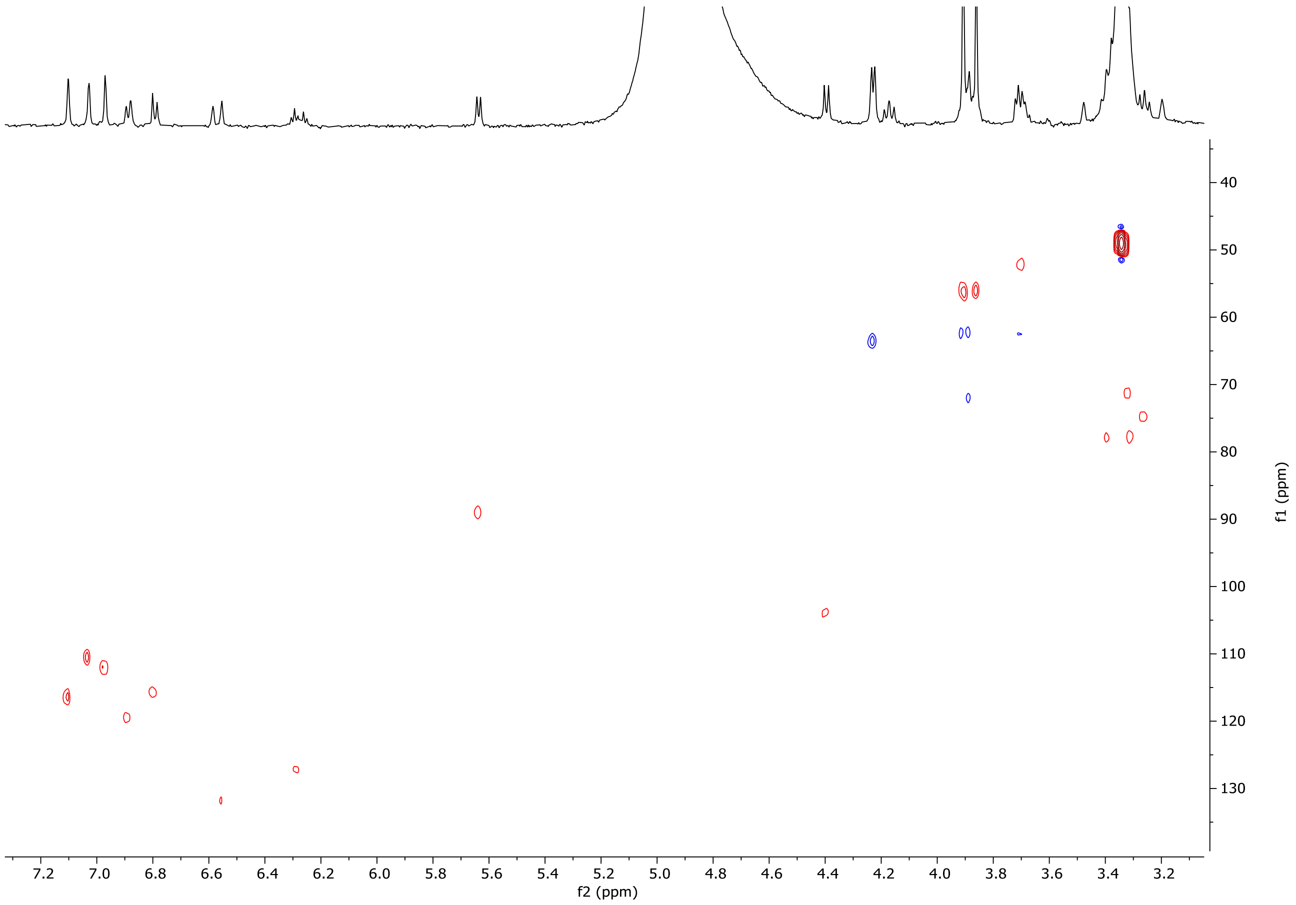

S20. HMBC (600 MHz, CD_3OD) of *dehydrodiconiferyl alcohol-9-O-β-D-glucopyranoside* (**7**).

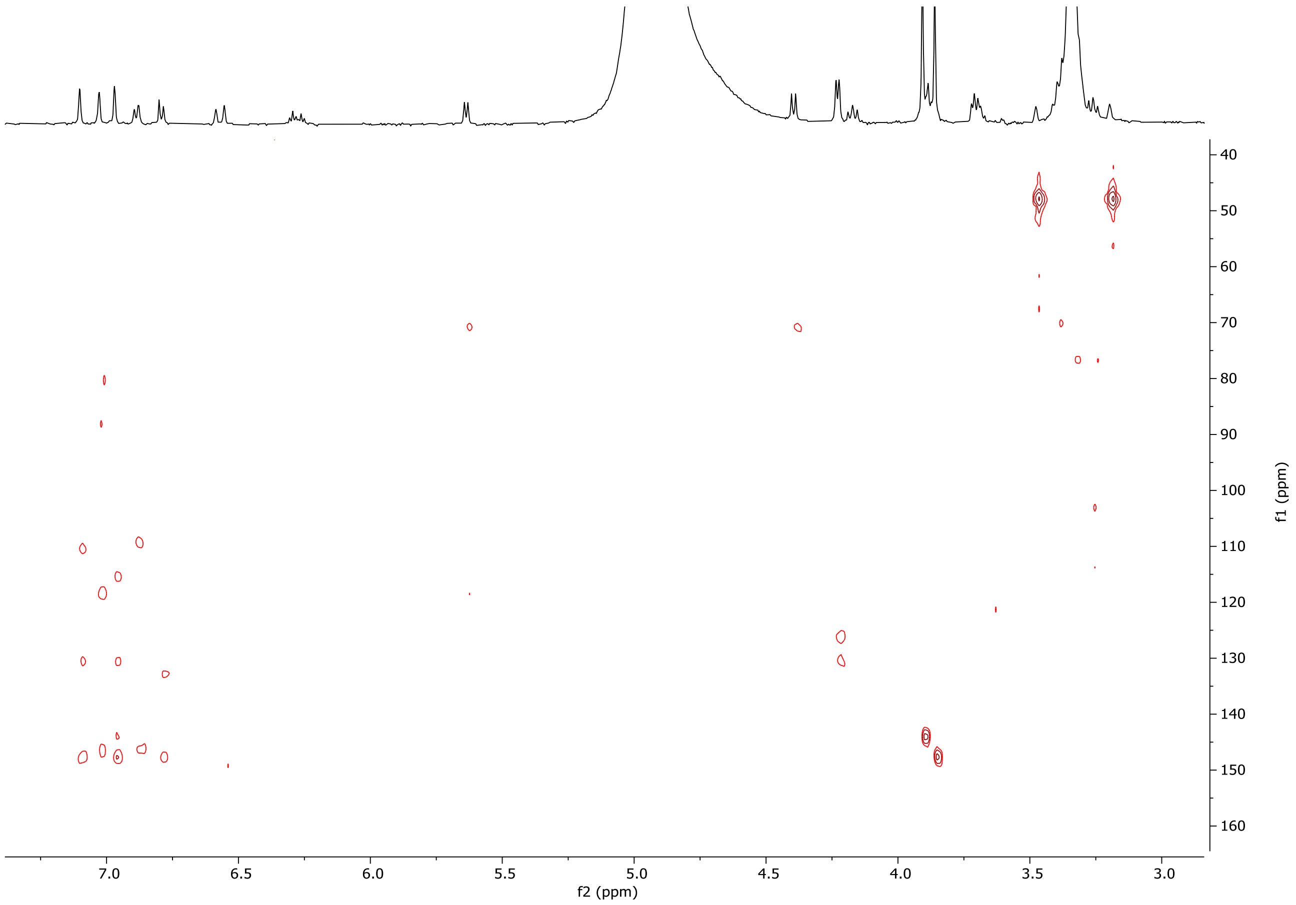

S21. ^{1}H-^{1}H COSY NMR (600 MHz, CD_3OD) of *dehydrodiconiferyl alcohol-9-O-β-D-glucopyranoside* (**7**).

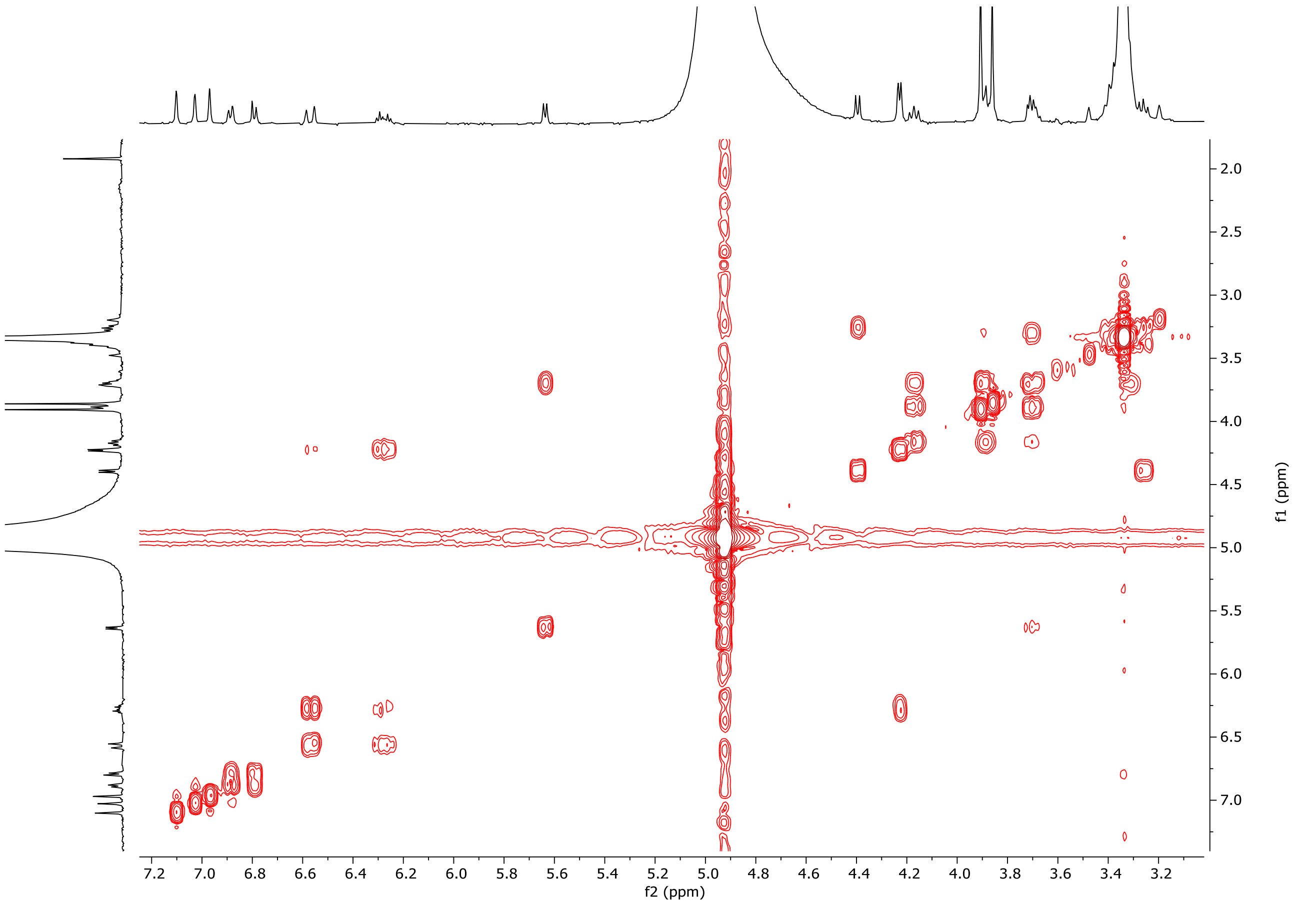

S22. ^{1}H NMR (600 MHz, CD_3OD) of *3,4,5-tri-O-methyl gallic acid* (**8**).

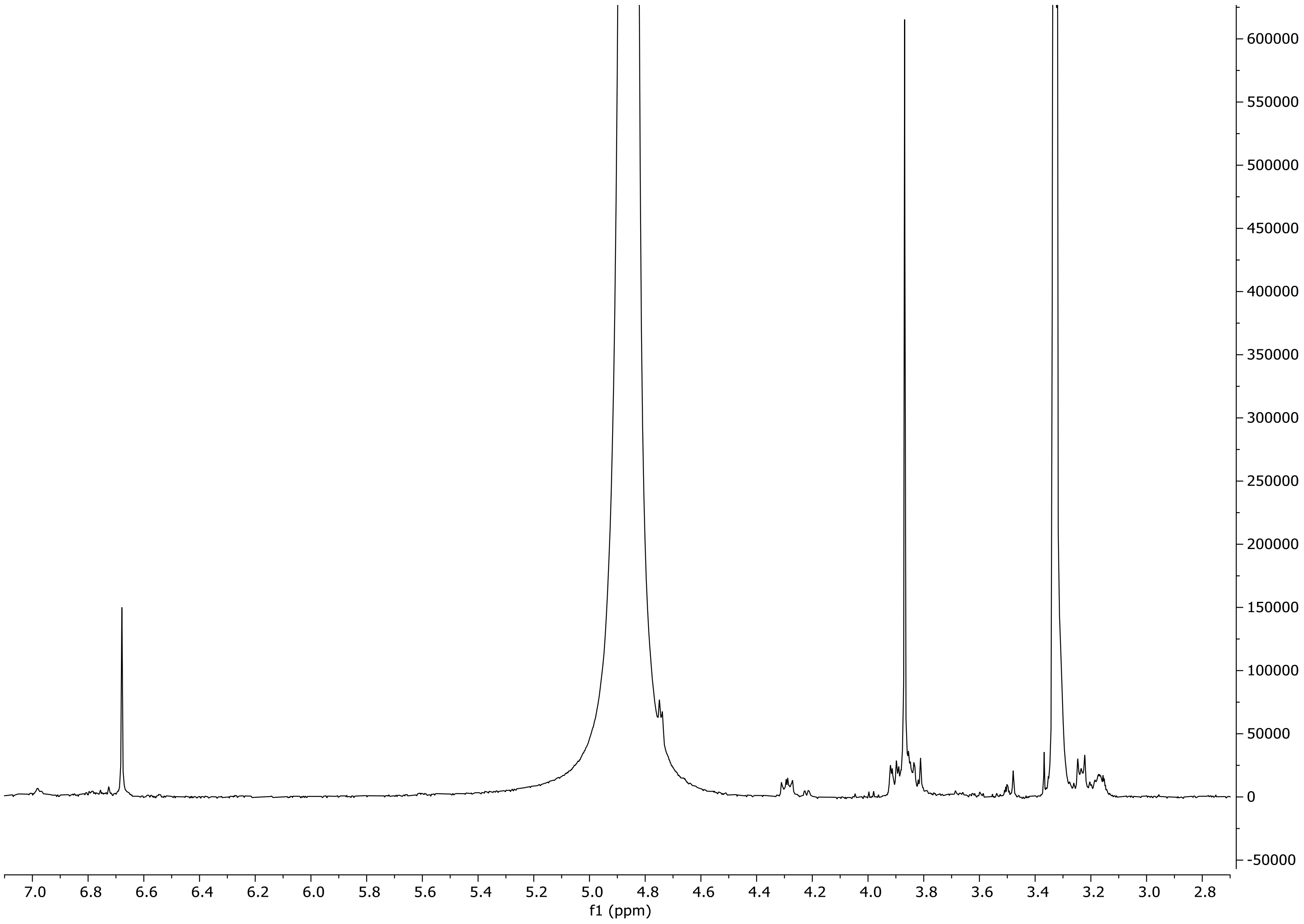

S23. HSQC (600 MHz, CD_3OD) of *3,4,5-tri-O-methyl gallic acid* (**8**).

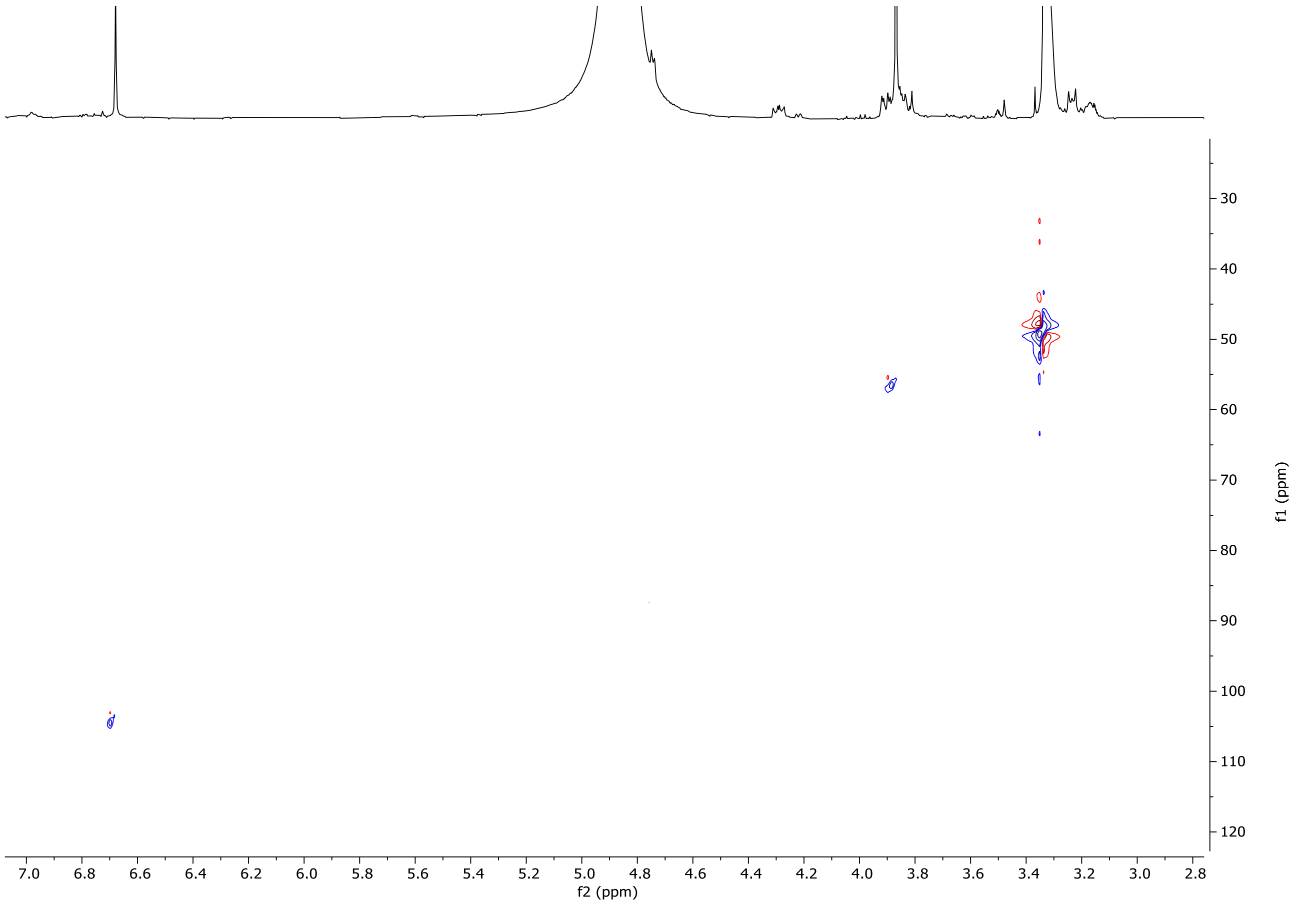

S24. ^{1}H NMR (600 MHz, CD_3OD) of (+)-l*ariciresinol* (**9**).

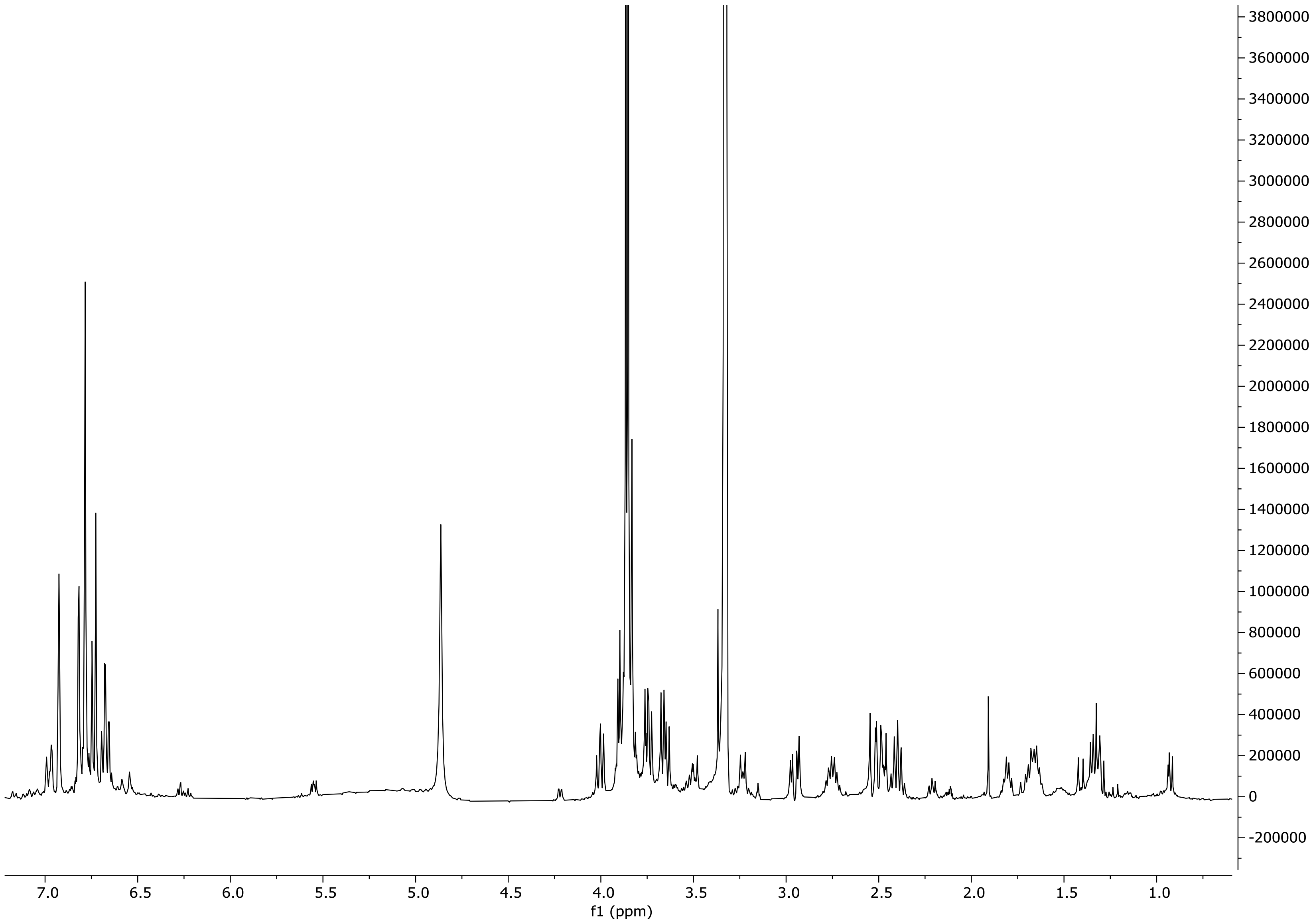

S25. HSQC (600 MHz, CD_3OD) of (+)-l*ariciresinol* (**9**).

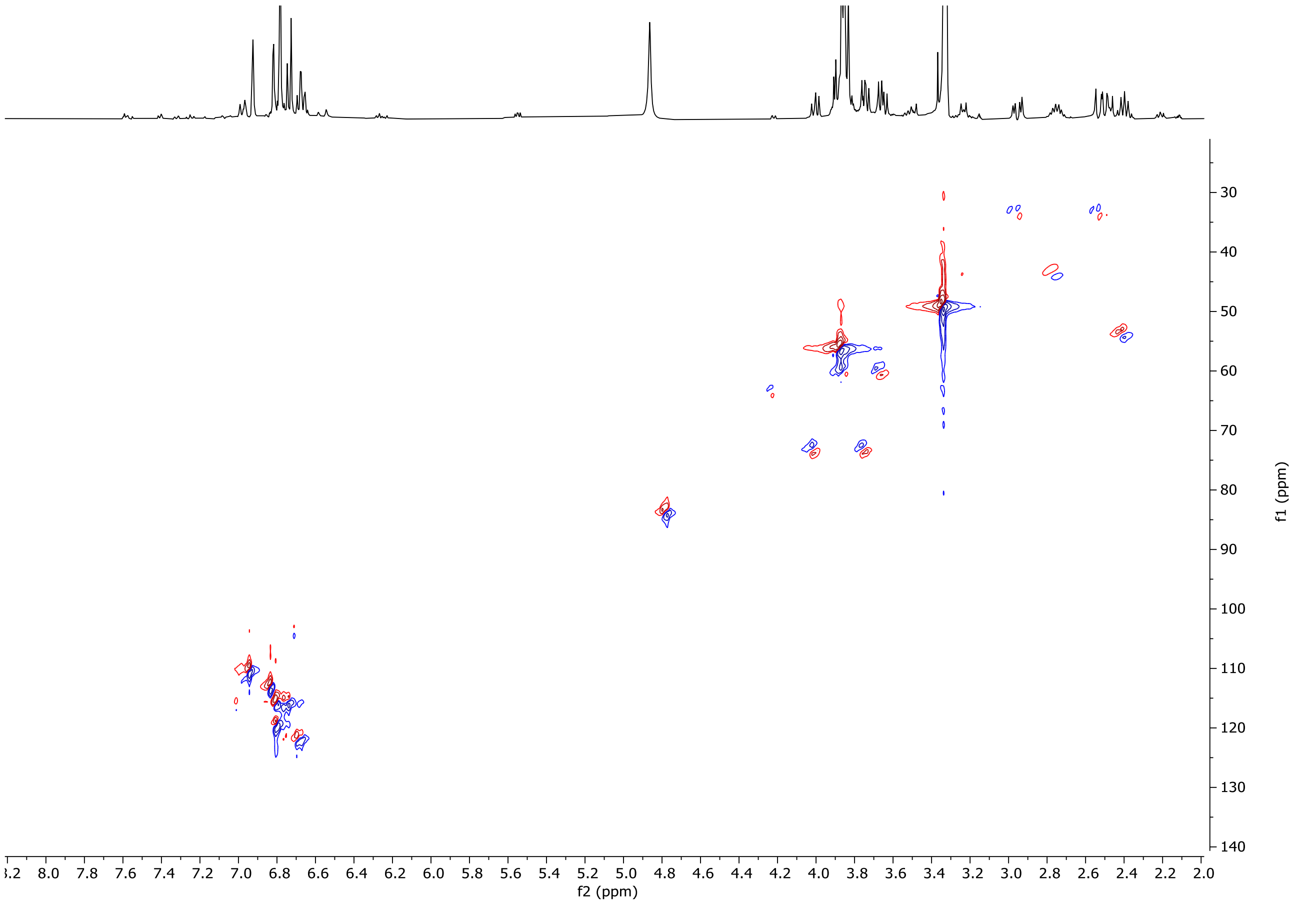

S26. ^{1}H NMR (600 MHz, CD_3OD) of *americanol A* (**10**).

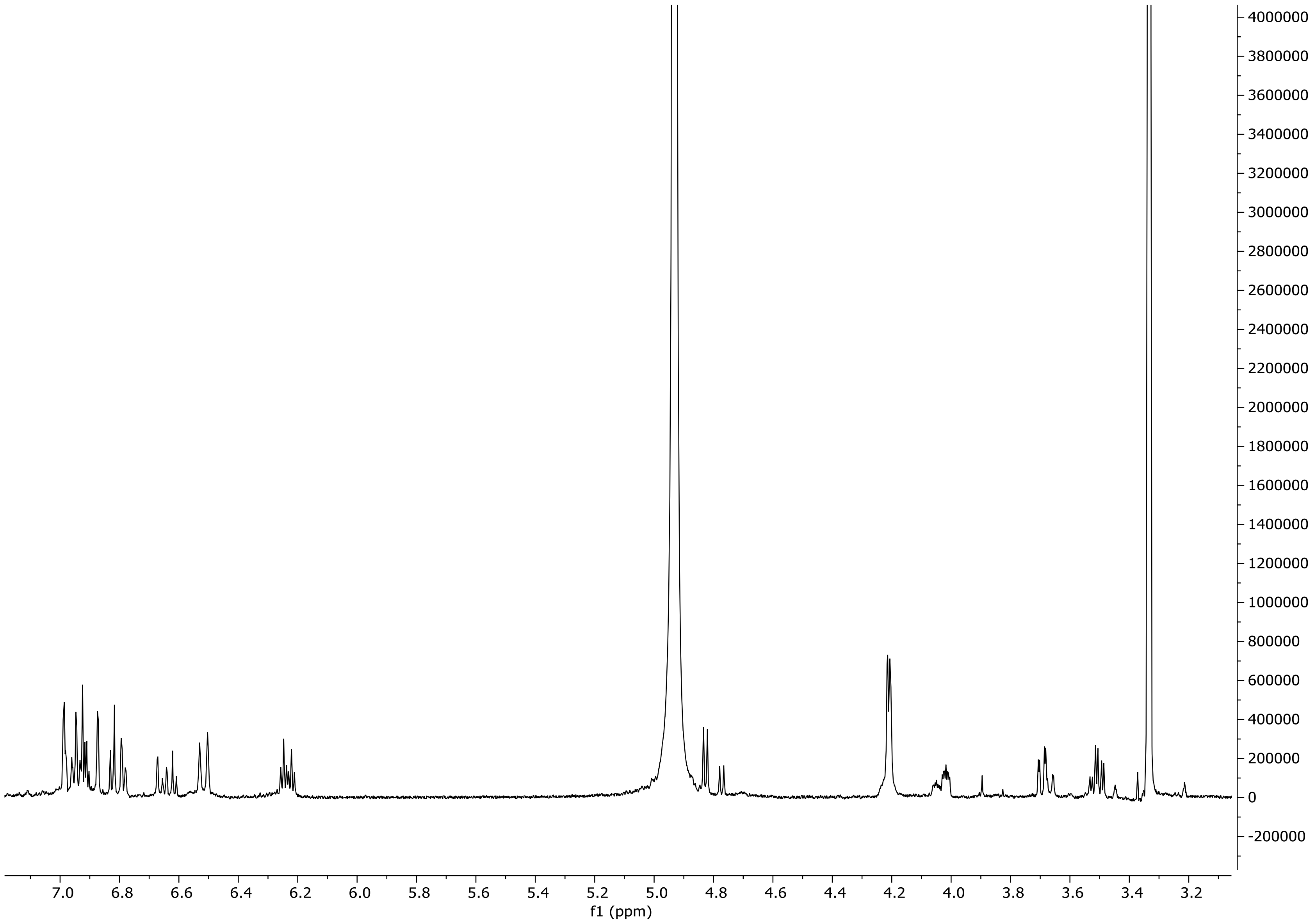

S27. HSQC (600 MHz, CD_3OD) of *americanol A* (**10**).

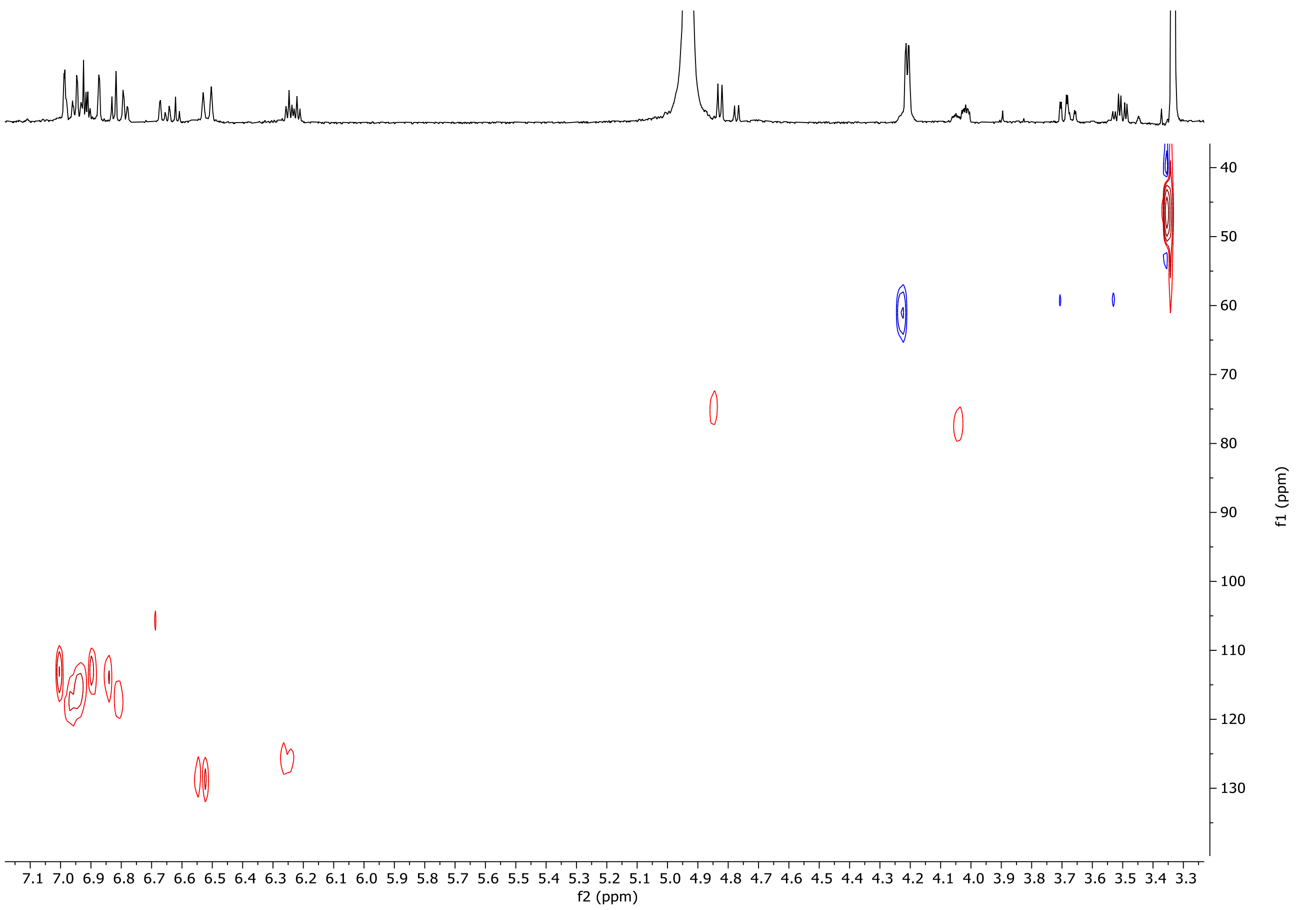

S28. HMBC (600 MHz, CD_3OD) of *americanol A* (**10**).

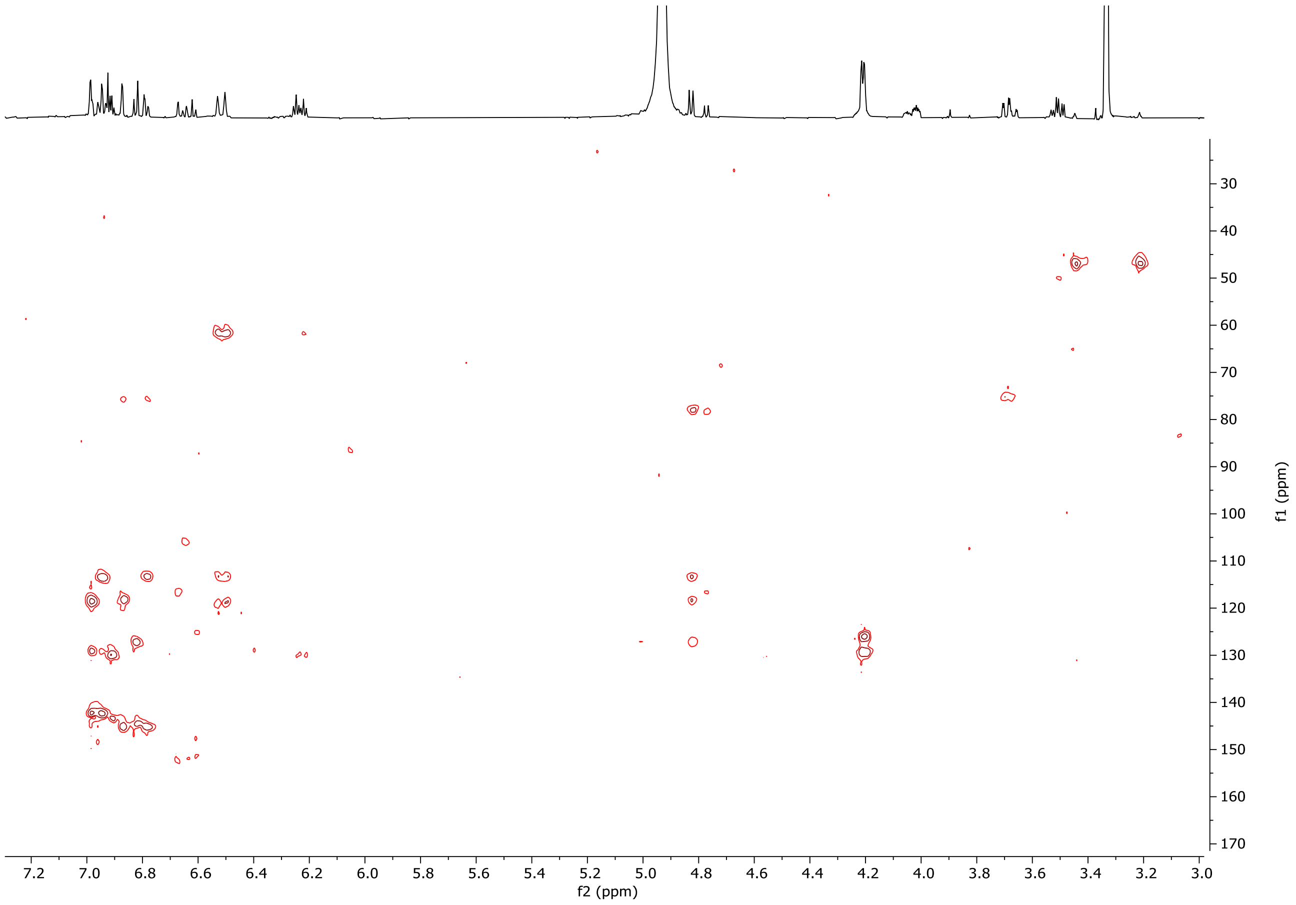

Supplement: Supplementary file 1 [file ijms-25-00252-s001.zip › ijms-2689048-supplementary.pdf]
